# Targeting DAP5 Disrupts Alternate Mode of Translational Initiation in Tregs and Potentiates Antitumor Immunity

**DOI:** 10.1002/advs.202520625

**Published:** 2025-12-28

**Authors:** Xiaojiang Lai, Guihong Pan, Xixi Feng, Jun Zhang, Yunxia Li, Shanmeizi Zhao, Zhenming Lin, Yun Feng, Wei Bao, Hongyan Yang, Chengmei Xie, Chen Huang, Jun Wang

**Affiliations:** ^1^ Precision Research Center for Refractory Diseases, State Key Laboratory of Innovative Immunotherapy Institute for Clinical Research Shanghai Key Laboratory of Pancreatic Diseases Department of Gastrointestinal Surgery Department of Gastroenterology Department of Nursing Department of Obstetrics and Gynecology Shanghai General Hospital Shanghai Jiao Tong University School of Medicine Shanghai 200080 China; ^2^ Department of Pediatric Surgery Guangdong Provincial Key Laboratory of Research in Structural Birth Defect Disease Guangdong Provincial Clinical Research Center for Child Health Guangzhou Institute of Pediatrics Guangzhou Women and Children's Medical Center Guangzhou Medical University Guangzhou 510623 China; ^3^ State Key Laboratory of Respiratory Disease the GMU‐GIBH Joint School of Life Sciences Guangzhou Medical University Guangzhou 511436 China; ^4^ Department of Reproductive Medicine Affiliated Jinling Hospital Medical School Nanjing University Nanjing 210002 China; ^5^ Reproductive Medicine Center Yuebei People's Hospital Shantou University Medical College Shaoguan 512026 China

**Keywords:** DAP5/eIF4G2, immune homeostasis, translation, Tregs, tumor microenvironment

## Abstract

Regulatory T cells (Tregs) can thrive in the harsh tumor microenvironment (TME) to dampen antitumor immunity. Chronic stresses within TME compromise canonical cap‐dependent translation (CDT), which compromises effector T cell function but not Treg persistence. Death‐associated protein 5 (DAP5/eIF4G2), a non‐classical translational scaffold, has been reported to support human Treg differentiation in vitro, but its functions in thymic Treg development, peripheral Treg homeostasis, and tumor‐infiltrating Treg (ti‐Treg) fitness remain unclear. Here, it is shown that DAP5 expression positively correlates with ti‐Treg frequencies in both colorectal cancer patients and murine subcutaneous tumors. Mice with homozygous Dap5 deletion in Tregs has intact thymic and peripheral Treg development but spontaneously developed typical scurfy symptoms. Haploinsufficiency of Dap5 in Tregs preserves peripheral immune homeostasis while suppressing tumor growth, with enhanced CD8^+^ T cell infiltration and effector function. Mechanistically, Dap5 mediates alternate mode of translation of transcripts encoding CD25 and MCL‐1 in Tregs, thereby sustaining Treg lineage stability and survival in the stressful TME. Overall, Tregs rely on DAP5‐driven alternate translation to maintain peripheral homeostasis and acquired fitness within the TME. Selective disruption of this pathway impairs ti‐Tregs while sparing systemic tolerance, offering a potential therapeutic strategy to enhance anti‐tumor immunity.

## Graphical abstract

Regulatory T cells (Tregs) suppress antitumor immunity. This study identifies that the translation scaffold DAP5/eIF4G2 is upregulated in tumor‐infiltrating Tregs (ti‐Tregs). DAP5 mediates an alternate translation mode to sustain CD25 and MCL‐1 expression, which is critical for ti‐Treg stability and survival in the tumor microenvironment. Selective disruption of DAP5 impairs ti‐Tregs and potentiates antitumor immunity.

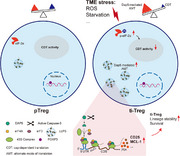

## 1 Background

Regulatory T cells (Tregs) are not only essential for self‐tolerance maintaining,^[^ ^1^ ^]^ but also actively contribute into establishment of immunosuppressed tumor microenvironment (TME) that dampens antitumor immunity.^[^ ^2^ ^]^ Targeting tumor‐infiltrating regulatory T cells (ti‐Tregs) is a promising strategy for cancer immunotherapy.^[^ ^3^ ^]^ However, targeting ti‐Tregs risks indiscriminate depletion of peripherally induced Treg (pTreg), potentially triggering lethal systemic autoimmune responses. Therefore, identifying ti‐Treg‐specific targets is essential to overcome this problem.

The mammalian target of rapamycin (mTOR) pathway integrates multiple signals including nutritional availability, cytokines and oxygen, to promote a cellular state characterized as high glycolytic metabolism and cap‐dependent translation (CDT) and rapid proliferation, thereby driving effector T cell (Teff) differentiation and activation,^[^ ^4^, ^5^, ^6^ ^]^ whereas mTOR activity in Tregs is fine‐tuned at lower level to maintain a distinct metabolic state as hypo‐glycolytic but hyper‐oxidative phosphorylation (OXPHOS) and fatty acid oxidation (FAO),^[^ ^7^, ^8^ ^]^ along with functional autophagy,^[^ ^9^ ^]^ thus preserving their immunosuppressive functions. Excessive mTOR activity can impair Treg differentiation and proliferation.^[^ ^10^, ^11^ ^]^ Additionally, ti‐Tregs can adapt to thrive in the harsh TME, which are detrimental to Teff.^[^ ^7^, ^12^, ^13^, ^14^ ^]^ While mTOR suppression is essential for Treg homeostasis, it may concurrently inhibit CDT, the primary protein synthesis pathway.^[^ ^15^ ^]^ This inhibition compromises global translation during Treg differentiation and functional maturation, therefore Tregs must employ alternative mechanisms for selective mRNA translation. How Tregs bypass the canonical translation machinery to achieve alternate mode of protein synthesis remains poorly understood.

The CDT is initiated by the recognition of the 7‐methylgluanosine (m^7^G) cap at the 5′ end of mRNAs by the classic eukaryotic initiation 4F complex (eIF4F), which comprises the scaffolding eIF4G1, the m^7^G cap binding protein eIF4E and the ATP‐dependent RNA helicase eIF4A.^[^ ^16^ ^]^ It is well‐acknowledged that CDT initiation of most eukaryotic mRNAs in the cytosol are largely halted when the eIF4F activity is compromised.^[^ ^17^ ^]^ This is a hallmark of the Integrated Stress Response (ISR), an adaptive mechanism to diverse stresses within TME. The ISR is manifested by eIF‐2α phosphorylation, which globally inhibits CDT while selectively translating mRNAs like ATF4 to orchestrate a survival‐oriented gene expression program.^[^ ^18^ ^]^

The underlying mechanisms that govern the translation of protein molecules essential for ti‐Treg survival in TME remain unclear. DAP5/eIF4G2, which lacks the N‐terminal region of eIF4G1, is unable to recruit the cap‐binding protein eIF4E but retains the ability to engage eIF4A and eIF3, thereby promoting the synthesis of proteins essential for cell survival by initiating alternate mode of translation (AMT).^[^ ^19^, ^20^, ^21^ ^]^ The roles of DAP5 in T cells remain unclear. Volta et al. reported that DAP5 is required for in vitro human iTreg differentiation,^[^ ^22^ ^]^ while DAP5 limits CD8^+^Teff function.^[^ ^23^ ^]^ Uncovering the in vivo functions of DAP5 in the development of thymic‐derived Tregs (tTregs), the homeostasis of pTregs and the acquired fitness of ti‐Tregs in TME is crucial for advancing our understanding of Treg biology and its translational applications in cancer immunotherapy. Additionally, the selective translatome directly initiated by DAP5 in Tregs remains to be elucidated.

In this study, we provided extensive in vitro and in vivo evidence demonstrating that pTreg homeostasis relies on DAP5‐mediated AMT. Mice with homozygous *Dap5* loss in Tregs (HO‐*Dap5^ΔFoxp3^*) exhibited intact tTreg development and pTreg differentiation, but they spontaneously developed fatal systemic autoimmune responses. Mice with single copy deletion of *Dap5* in Tregs (HE‐*Dap5^ΔFoxp3^*) retained pTreg homeostasis under steady state, but failed to sustain ti‐Treg stability and survival in TME wherein ISR is activated in ti‐Tregs, as HE‐*Dap5^ΔFoxp3^* Tregs were defective in Dap5‐mediated selective translation of molecules (such as CD25 and MCL‐1) that are indispensable for their stability and survival in TME. Our findings revealed that acquired fitness of ti‐Tregs rely on DAP5‐mediated AMT and targeting DAP5 is of important therapeutic potential in cancer treatment.

## 2 Results

### 2.1 DAP5 is Associated with ti‐Treg Abundance in Human Cancers

**Figure 1:**
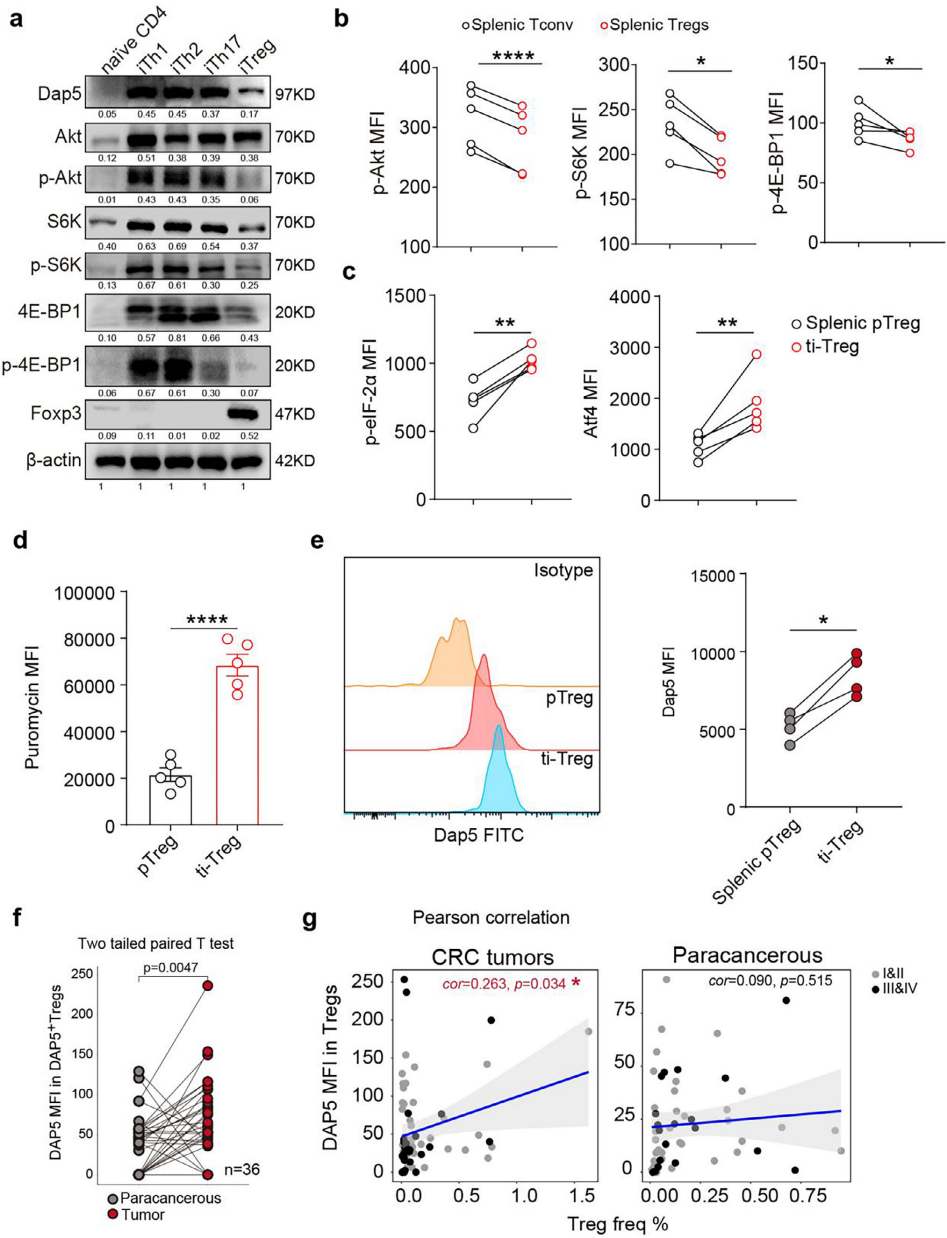
Elevated DAP5 expression in ti‐Tregs is associated with ti‐Treg infiltrations. a) Western blotting results comparing activities of AKT/mTOR signaling pathway across naïve CD4^+^ T, iTh1, iTh2, iTh17 and iTregs. b) Flow cytometric results confirming reduced phosphorylation levels of Akt, S6K and 4E‐BP1 in mouse splenic Tregs than in conventional T cells (Tconv). c) Flow cytometric results revealing increased mean fluorescent intensities (MFI) of p‐eIF‐2α and Atf4 in ti‐Tregs than in pTregs. d) Box plots displaying higher puromycin‐labeling intensities among ti‐Tregs than in pTregs. e) Left panel: the representative histograms depicting distribution of Dap5 expressions among pTregs and ti‐Tregs; right panel: dot plots showing pairwise comparison of Dap5 MFI between ti‐Tregs and pTregs. f) The MFI of DAP5 among tiTregs compared with that in Tregs from paracancerous tissues. The *p*‐value was determined by performing two‐tailed paired T test. g) Scatter plot displaying positive correlation between the DAP5 MFI and ti‐Treg frequencies in CRCs. *p‐*values were determined by two‐tailed paired Student's *T*‐test (b, c, e and f), unpaired Student's *T*‐test (d) and Spearman's correlation Test (g), ^*^ *p*<0.05, ^**^ *p*<0.01, ^***^ *p*<0.001, ^****^ *p*<0.0001.

Wondering whether translation mode in Tregs is distinct from CD4^+^ Teff subpopulations, we harvested in vitro induced Tregs (iTregs), iTh1, iTh2 and iTh17 derived from mouse naïve CD4^+^ T cells and evaluated their activity of AKT/mTORC1 pathway which is essential for canonical CDT. Indeed, iTregs displayed hypo‐phosphorylation levels of Akt, ribosomal protein S6 kinase (S6K) and eukaryotic translation initiation factor 4E binding protein 1 (4E‐BP1) compared with naïve CD4^+^ T, iTh1, iTh2 and iTh17 (**Figure** **1**a; Figure S1a, Supporting Information). Flow cytometric results confirmed significantly reduced levels of p‐Akt, p‐S6K and p‐4E‐BP1 in Tregs than in conventional CD4^+^ T cells (Tconv) in spleens (Figure 1b; Figure S1b, Supporting Information). These results suggested that CDT pathway in pTregs were more repressed than in pTconv, as S6K and 4E‐BP1 are the main targets of the AKT/mTORC1 and their phosphorylation levels are crucial for the initiation of CDT.^[^ ^24^ ^]^

In C57BL/6 mice with subcutaneous tumor growth, ti‐Tregs exhibited significantly elevated p‐eIF‐2α and Atf4 expression (Figure 1c), indicating that ti‐Tregs exhibited enhanced ISR level. In eukaryotic cells, the ISR normally leads to a global shutdown of protein translation.^[^ ^18^ ^]^ We next compared translation activity between ti‐Tregs and pTregs. Puromycin terminates translation by mimicking aminoacyl‐tRNA and occupying the ribosomal A‐site.^[^ ^25^ ^]^ This results in covalent attachment of puromycin to the C‐terminal of nascent polypeptides, enabling quantification of cellular translation activity through immunodetection of puromycin incorporation.^[^ ^26^ ^]^ Unexpectedly, ti‐Tregs exhibited significantly higher puromycin labeling intensity than splenic pTregs (Figure 1d). Meanwhile, ti‐Tregs had markedly higher expression of Dap5 (Figure 1e). We speculated that ti‐Tregs rely more heavily on Dap5‐mediated AMT than pTregs for stronger protein translational activity.

We subsequently focused on the function of DAP5 to elucidate the importance of AMT for ti‐Tregs. To gain clinical relevance of DAP5 expression to ti‐Treg fitness in TME, we performed data mining with RNA sequencing data from varieties of human cancers on the web server GEPIA2 (http://gepia2.cancer‐pku.cn/).^[^ ^27^ ^]^ We observed that *DAP5* expression and effector ti‐Treg signature (ti‐eTregs) (*FOXP3*, *CTLA4*, *CCR8* and *TNFRSF9*) exhibited a significantly positive correlation across various human tumor samples (Figure S1c, Supporting Information), however, this association may be partially influenced by DAP5 expression in other cell types within tumors. We further gathered a cohort of patients with colorectal cancers (CRCs) (Table S1, Supporting Information). Results of multicolor immunofluorescence (mIF) revealed that the mean fluorescent intensities (MFI) of DAP5 in ti‐Tregs were significantly higher compared to Tregs in paracancerous region (Figure 1f; Figure S1d, Supporting Information). We also observed a positive correlation (*cor*=0.263, *p*=0.034) between Treg frequencies and intracellular DAP5 expression levels among CRC tumors rather than paracancerous tissues (Figure 1g).

### 2.2 Mice with Homozygous Dap5 Ablation in Tregs Exhibited Normal tTreg Development and pTreg Differentiation In Vivo

To dissect the function of Dap5 in Tregs in vivo, we generated transgenic mice with specific *Dap5* ablation in Tregs (*Dap5^ΔFoxp3^*) by crossing the floxed‐*Dap5* mice with the *Foxp3*‐*Cre* (B6.129(Cg)‐*Foxp3^4(YFP/icre)Ayr^*/J) strain from Jackson Laboratory that are widely used in Treg studies (Figure S2a, Supporting Information). Since *Foxp3* is located on the X chromosome and considering the phenomenon of X chromosome inactivation in female mice, we designated *Dap5^fl/fl^* ×*Foxp3^Cre/Cre^* females and *Dap5^fl/fl^* ×*Foxp3^Cre^* males as homozygous knockout (HO‐*Dap5^ΔFoxp3^*) hereafter to ensure equal expression of Cre recombinase expression in both genders. Similarly, the *Dap5^fl/+^* ×*Foxp3^Cre/Cre^* females and *Dap5^fl/+^* ×*Foxp3^Cre^* males were termed as heterozygous knockout (HE‐*Dap5^ΔFoxp3^*).

**Figure 2:**
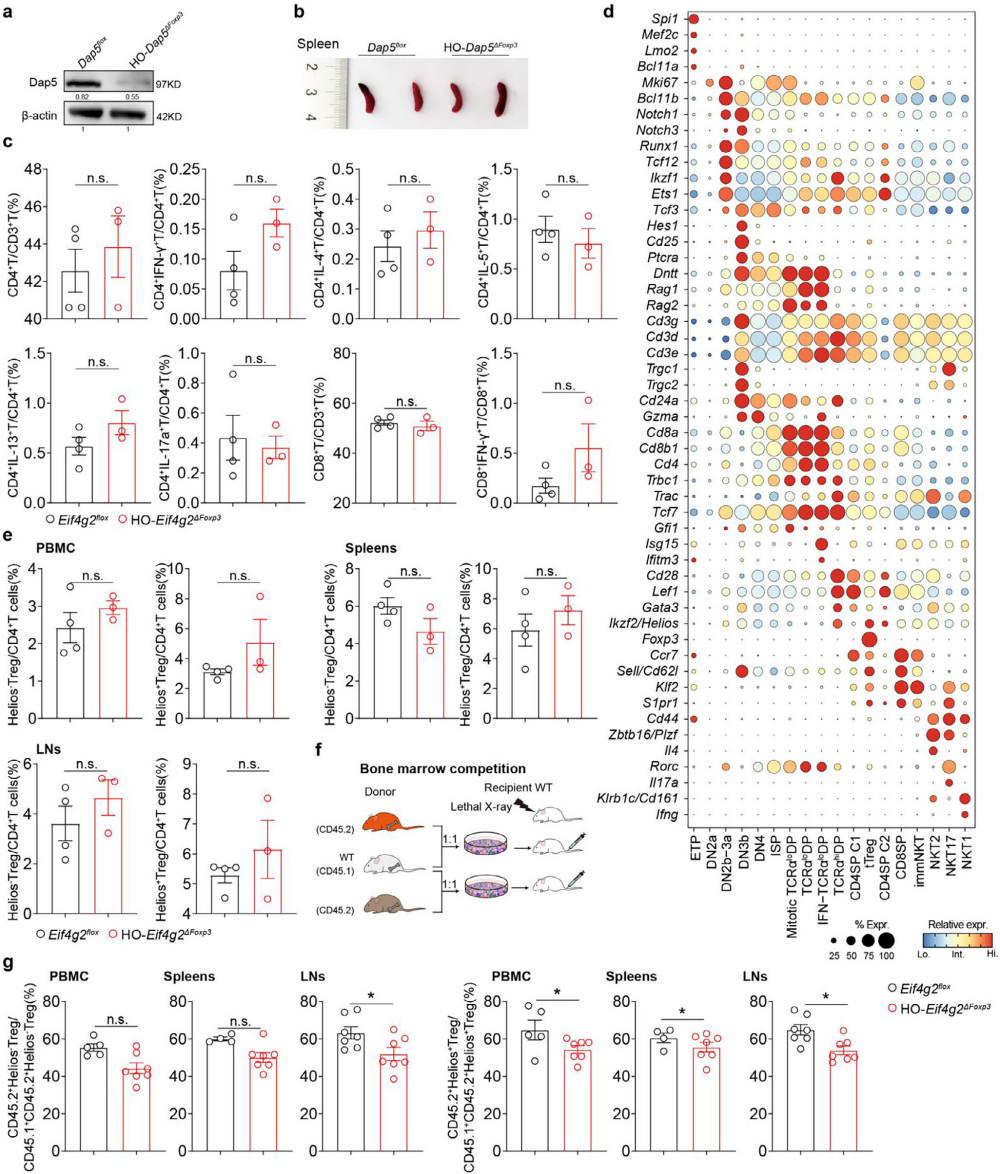
Ablation of Dap5 in Tregs did not affect tTreg development and pTreg differentiation in vivo. a) Naïve CD4^+^ T cells were purified from 4‐weeks‐old *Dap5^flox^* and age‐matched *HO‐Dap5^ΔFoxp3^* mice and differentiated into iTreg in vitro. The absence of Dap5 protein expression in iTregs from *HO‐Dap5^ΔFoxp3^* mice was assessed by performing WB. b) Similar spleen sizes between 4‐weeks‐old *Dap5^flox^* and age‐matched *HO‐Dap5^ΔFoxp3^* mice. c) Bar plots showing unchanged proportions of indicated T cell subpopulations in the PBMC from 4‐weeks‐old *HO‐Dap5^ΔFoxp3^* and age‐matched *Dap5^flox^* mice. d) Dot plots displaying expression levels of the canonical marker genes across different thymocyte subpopulations. e) Bar plots showing unaltered proportions of Helios^−^ Treg and Helios^+^ Treg in PBMCs, spleen and lymph nodes between 4‐weeks‐old *HO‐Dap5^ΔFoxp3^* and age‐matched *Dap5^flox^* mice. f) Schematic diagram depicting the workflow of bone morrow (BM) chimera experiment. Briefly, the BM cells were isolated from age‐matched and sex‐matched *Dap5^flox^* (CD45.2), *HO‐Dap5^ΔFoxp3^* (CD45.2) and wild type (WT) C57BL/6 (CD45.1). The BM cells from *HO‐Dap5^ΔFoxp3^* or *Dap5^flox^* mice were mixed with BM cells from WT (CD45.1) mice at 1:1 ratio and intravenously injected into lethally irradiated (9 Gy) WT C57BL/6 recipient mice for subsequent bone marrow reconstitution. g) Bar plots comparing the contributions of Helios^+^ Tregs and Helios^−^ Tregs derived from donors of *Dap5^flox^* and *HO‐Dap5^ΔFoxp3^* mice in the BM‐reconstituted recipients. *p‐*values were determined by two‐tailed student's *T*‐test (c, e and g), ^*^ *p*<0.05.

Western blotting detection demonstrated loss of Dap5 expression in iTregs from *Dap5^ΔFoxp3^* mice (**Figure** **2**a). The self‐tolerance of homozygous mice (HO‐*Dap5^ΔFoxp3^*) was normal as control mice (*Dap5^flox^*) up to 5 weeks of age (similar spleen sizes, Figure 2b) and no significant changes of Teff frequencies in PBMC, spleen and lymph nodes were observed (Figure 2c; Figure S2b,c, Supporting Information). We performed single cell RNA sequencing (scRNA‐seq) with thymocytes from HO‐*Dap5^ΔFoxp3^* and control mice (≈4 weeks old). Intact thymic T cell development spectrum from early T cell progenitors (ETPs) to double negative thymocytes (DNs), double positive (DPs) and single positive thymocytes (SPs) was captured and tTregs were distributed in scattered manner within CD4SP in both HO‐*Dap5^ΔFoxp3^* and control mice (Figure 2d; Figure S2d and Table S2, Supporting Information). The frequencies of the thymic T cell subpopulations including tTregs were unaltered in HO‐*Dap5^ΔFoxp3^* mice compared with *Dap5^flox^* mice (**Table** **1**). Transcriptomic comparison of tTregs between HO‐*Dap5^ΔFoxp3^* and control mice revealed none differentially expressed genes (data not shown). Flow cytometric results confirmed that HO‐*Dap5^ΔFoxp3^* mice (≈4 weeks old) had equal abundances of Helios^+^ Tregs and Helios^−^ Tregs in PBMC, spleens, lymph nodes and thymus (Figure 2e; Figure S2e, Supporting Information). We performed competitive bone marrow transplantation (BMT) experiments by transferring 1:1 mixed BM from wild type mice (WT, CD45.1) with HO‐*Dap5^ΔFoxp3^* (CD45.2) or *Dap5^flox^* mice (CD45.2) into BM‐deprived WT mice (Figure 2f; Figure S2f, Supporting Information). Following BM reconstitution, we did not observe dramatic changes in frequencies of peripheral Helios^+^ Tregs and Helios^−^ Tregs originating from *Dap5^flox^*‐BM (CD45.2) and HO‐*Dap5^ΔFoxp3^*‐BM (CD45.2), respectively, in the corresponding BM‐reconstituted recipient mice (Figure 2g), although the peripheral frequencies of Tregs derived from HO‐*Dap5^ΔFoxp3^*‐BM (CD45.2) were slightly lower, possibly due to survival defects. These results collectively indicated that ablation of Dap5 in Tregs did not impair tTreg development and pTreg differentiation in vivo.

**Table 1:** Cell numbers of thymocyte subpopulations from *HO‐Dap5^ΔFoxp3^* and *Dap5^flox^* mice at the age of 4 weeks.

Cluster	*Dap5^flox^*		*HO‐Dap5^ΔFoxp3^*		Markers
	M45	M64	M49	M40	
ETP	64 (1.50%)	68 (1.17%)	46 (0.87%)	83 (1.68%)	*Spi1* ^+^ *Bcl11a* ^+^ *Lmo2* ^+^
DN2b	29(0.68%)	38(0.66%)	25(0.47%)	17(0.34%)	*Bcl11b* ^lo^ *Notch1* ^lo^ *Mki67* ^+^
DN3a	48(1.13%)	78(1.35%)	62(1.17%)	44(0.89%)	*Bcl11b* ^hi^ *Notch1* ^hi^ *Mki67* ^hi^ *Rag1* ^+^
DN3b	116(2.73%)	106(1.83%)	127(2.40%)	112(2.27%)	*Bcl11b* ^int^ *Notch1* ^hi^ *Mki67* ^lo^ *Rag1* ^+^ *Dntt* ^+^ *Ptcra* ^+^ *Il2ra* ^hi^
DN4	284(6.68%)	234(4.04%)	274(5.19%)	249(5.04%)	*Cd44* ^−^ *Cd25* ^lo/−^ *Cd28* ^+^
ISP	410(9.64%)	398(6.87%)	429(8.12%)	371(7.52%)	*Cd28* ^+^ *Cd4* ^−^ *Cd8* ^+^
Mitotic TCRα^lo^DP	305(7.17%)	268(4.63%)	259(4.90%)	252(5.11%)	*Mki67* ^+^ *Cd4* ^+^ *Cd8a* ^+^ *Cd8b1* ^+^ *Cd28* ^+^ *Rag2* ^hi^
TCRα^lo^DP	1730(40.67%)	3151(54.41%)	2846(53.88%)	2318(46.96%)	*Cd4* ^+^ *Cd8a* ^+^ *Cd8b1* ^+^ *Cd28* ^+^ *Trac* ^lo^ *Rag2* ^hi^
IFN‐TCRα^lo^DP	15(0.35%)	20(0.35%)	9(0.17%)	90(0.18%)	*Cd4* ^+^ *Cd8a* ^+^ *Cd8b1* ^+^ *Cd28* ^+^ *Trac* ^lo^ *Isg15* ^+^ *Rag2* ^hi^
TCRα^hi^DP	404(9.50%)	551(9.51%)	493(9.33%)	461(9.34%)	*Cd4* ^+^ *Cd8a* ^+^ *Cd8b1* ^+^ *Cd28* ^+^ *Trac* ^hi^ *Rag2* ^lo^
CD4SP C1	460(10.81%)	507(8.75%)	425(8.05%)	580(11.75%)	*Cd4* ^+^ *Cd8a* ^−^ *Cd8b1* ^−^ *Ccr7* ^+^
**tTreg**	**36(0.85%)**	**40(0.69%)**	**23(0.44%)**	**44(0.89%)**	*Foxp3* ^+^ *Ikzf2/Helios* ^+^ *S1pr1* ^+^
CD4SP C2	93(2.19%)	109(1.88%)	96(1.82%)	110(2.23%)	*Cd4* ^+^ *Cd8a* ^−^ *Cd8b1* ^−^ *Ccr7* ^lo^
CD8SP	139(3.27%)	130(2.24%)	96(1.82%)	160(3.24%)	*Cd4* ^−^ *Cd8a* ^+^ *Cd8b1* ^+^ *S1pr1* ^+^
immNKT	79(1.86%)	67(1.16%)	40(0.76%)	79(1.60%)	*Cd24a* ^−^ *Ccr7* ^+^
NKT2	18(0.42%)	8(0.14%)	8(0.15%)	5(0.10%)	*Zbtb16/Plzf* ^hiI^ *Il4* ^+^
NKT17	5(0.12%)	8(0.14%)	5(0.09%)	5(0.10%)	*Rorc* ^+^ *Plzf* ^intI^ *Il17a* ^+^
NKT1	19(0.45%)	10(0.17%)	19(0.36%)	37(0.75%)	*Plzf* ^lo^ *Ifng* ^+^
***N***	**4254**	**5791**	**5282**	**4936**	

**Note**: ETP: Early T cell progenitors

DN: Double negative thymocytes

ISP: Immature single (CD8^+^) positive thymocytes

SP: Single positive thymocytes

immNKT: immature natural killer T cell

To infer the different importance of Dap5 in pTreg homeostasis versus tTreg development, we next detected the mTORC1 signaling in tTreg precursors. The flowcytometric results showed that thymic Foxp3^+^CD4SPs had significantly higher levels of p‐Akt, p‐4E‐BP1 and p‐S6K but unchanged Dap5 expression compared to the Foxp3^−^CD4SPs (Figure S2g, Supporting Information), suggesting that thymic Treg progenitors possess significant mTORC1 signaling activities and may rely on canonical CDT rather than Dap‐5 mediated AMT.

### 2.3 Tregs with Homozygous Deletion of Dap5 Displayed Survival Defects in the Periphery

**Figure 3:**
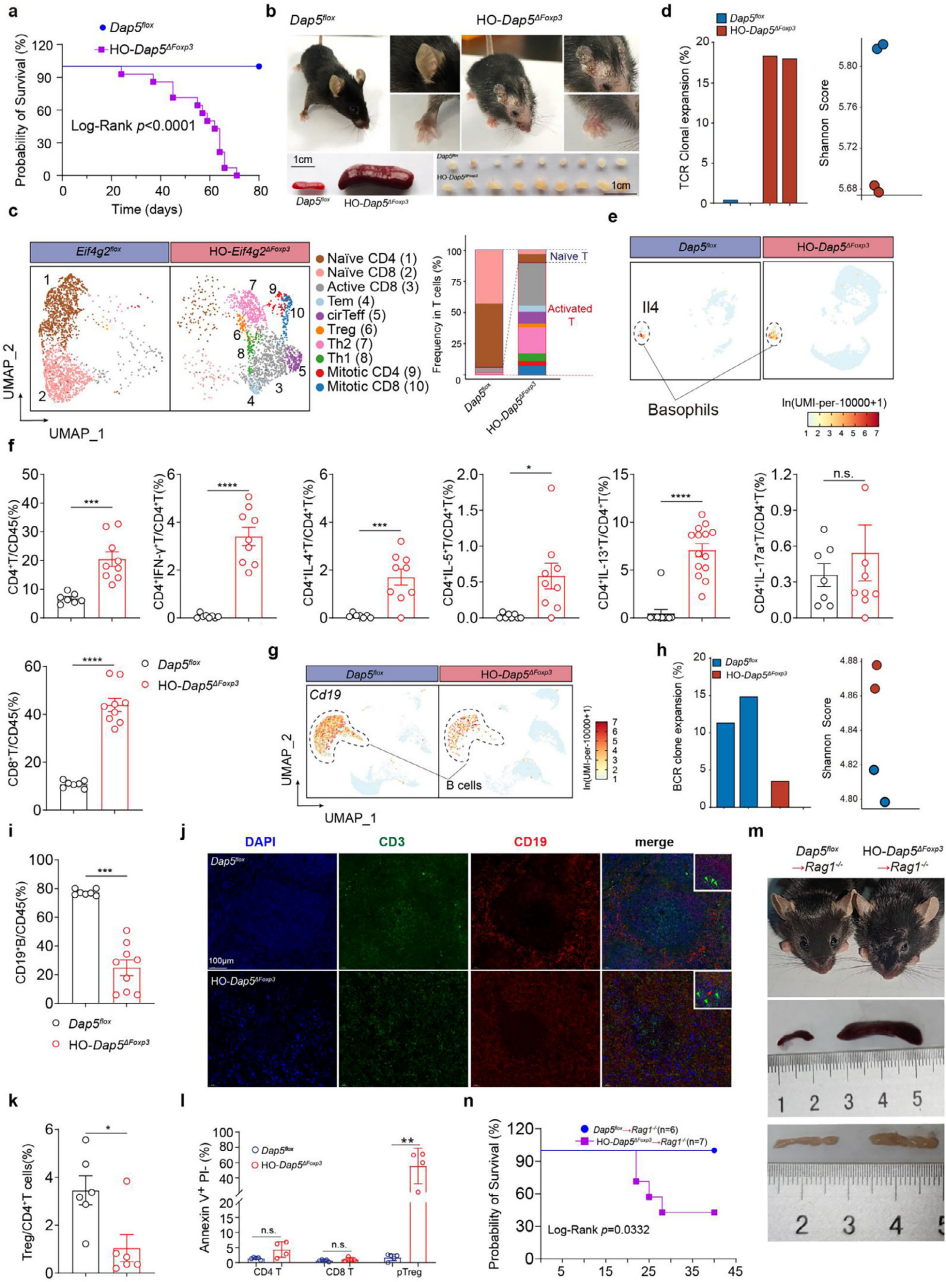
HO‐*Dap5^ΔFoxp3^* mice displayed lethal autoimmune tolerance defects. a) Under the same housing condition, the lifespan of HO‐*Dap5^ΔFoxp3^* mice were significantly shortened compared with *Dap5^flox^* littermates. b) Around 6‐8 weeks‐old, HO*‐Dap5^ΔFoxp3^* mice had enlarged lymphoid organs, scaly patches on the skin tissues of the head and ears, hair loss, and swollen toes. c) **Left panel**: 2D uniform manifold approximate projection (2D‐UMAP) plots comparing the distribution of T cell subpopulations in PBMCs between 6‐weeks‐old *Dap5^flox^* and HO‐*Dap5^ΔFoxp3^* mice; **right panel**: stacked bar plots comparing the compositions of naïve T cells and effector T cells between *Dap5^flox^* and HO‐*Dap5^ΔFoxp3^* mice. d) Enhanced TCR clonal expansion (left) and reduced Shannon diversity indexes of TCRs (right) in the peripheral T cells from HO‐*Dap5^ΔFoxp3^* mice compared with that from *Dap5^flox^* mice. e) 2D‐UMAP plots showing elevated presence of *Il4* ^+^ basophils in the peripheral blood of HO‐*Dap5^ΔFoxp3^* mice. f) Bar plots comparing the frequencies of indicated immune cell populations in the peripheral blood from 6‐weeks‐old *HO‐Dap5^ΔFoxp3^* mice and age‐matched *Dap5^flox^* mice. g) 2D‐UMAP plots displaying reduced B cell presence in the peripheral blood of *HO‐Dap5^ΔFoxp3^* mice. h) Reduced BCR clonal expansion (left) and increased Shannon diversity indexes of BCR (right) in the peripheral B cells from HO‐*Dap5^ΔFoxp3^* mice compared with that from *Dap5^flox^* mice. i) The bar plot demonstrates a reduction of peripheral B cells in *HO‐Dap5^ΔFoxp3^* mice relative to the age‐matched *Dap5^flox^* mice. j) The multi‐color immunofluorescence (mIF) pictures showing impaired GC architectures and reduced B cells numbers in the spleens from 6‐weeks‐old *HO‐Dap5^ΔFoxp3^* mice compared with that from age‐matched *Dap5^flox^* mice. DAPI (blue), CD3 (green), CD19 (red). k) bar plot indicating decreased frequencies of peripheral Treg cells in *HO‐Dap5^ΔFoxp3^* mice compared to *Dap5^flox^* mice. l) Bar plots showing pTregs, but not CD4^+^ T or CD8^+^ T cells, derived from derived from *HO‐Dap5^ΔFoxp3^* mice were apoptotic (PI^+^ annexin V^+^). m) Naïve CD4^+^ T cells from *Dap5^flox^* or *HO‐Dap5^ΔFoxp3^* mice were intravenously injected into *Rag1^−/−^* mice. Approximately 20 days later, enlarged spleens and lymph nodes were observed in *HO‐Dap5^ΔFoxp3^* mice. n) The survival curve showing that the lifespan of *Rag1* ^−/−^ mice receiving intravenous injection of naïve CD4^+^ T cells from *HO‐Dap5^ΔFoxp3^* mice was significantly shortened. *p‐*values were determined by Log‐rank test (a and n), or two‐tailed student's *T*‐test (f, g, h, i, k and l), ^*^ *p*<0.05, ^**^ *p*<0.01, ^***^ *p*<0.001, ^****^ *p*<0.0001.

The HO‐*Dap5^ΔFoxp3^* mice spontaneously developed severe systemic autoimmune symptoms at the age ≈ 6 weeks and died soon (**Figure** **3**a). The sick HO‐*Dap5^ΔFoxp3^* mice displayed typical scurfy phenotypes, such as significantly enlarged lymphoid organs, scaly patches on the head and ear skin, dermal inflammation, hair loss, swollen toes (Figure 3b). We also conducted H&E staining on the skin, ears, and footpads of HO‐*Dap5^ΔFoxp3^* mice and observed a significant increase in inflammatory cell infiltration in multiple tissues and organs (Figure S3a, Supporting Information). These results indicate that the conditional deletion of HO‐*Dap5^ΔFoxp3^* in Tregs leads to immune dysregulation in mice.

To elucidate the disrupted immune status in the HO‐*Dap5^ΔFoxp3^* sick mice, we profiled peripheral immune status for the sick HO‐*Dap5^ΔFoxp3^* by performing scRNA‐seq with peripheral blood mononuclear cells (PBMC) (Figure S3b, Supporting Information). Majority of the T cells in the PBMC from HO‐*Dap5^ΔFoxp3^* mice were activated T cells including Th1, Th2, activated CD8^+^ T cells (active CD8^+^ T), circulating effector T cells (cirTeff), effector memory T cells (Tem), and *Ifng* ^+^ *Mki67* ^+^ proliferating CD4^+^ and CD8^+^ T cells, while T cells from age‐matched *Dap5^flox^* mice were mainly naïve T cells (Figure 3c; Figure S3c and Table S3, Supporting Information). T cells from HO‐*Dap5^ΔFoxp3^* mice were clonally expanded (Figure 3d; Figure S3d, Supporting Information). Regarding to myeloid compartment, more basophils (*Il1rl1* ^+^ *Mcpt8* ^+^ *Il3ra* ^+^) expressing *Il4* were present in the peripheral blood of HO‐*Dap5^ΔFoxp3^* mice (Figure 3e; Figure S3e,f and Table S4, Supporting Information).

Flow cytometric results confirmed that HO‐*Dap5^ΔFoxp3^* mice showed significantly increased abundance of IFN‐γ^+^CD4^+^ T cells and IFN‐γ^+^CD8^+^ T cells, IL‐4^+^/IL‐13^+^ CD4^+^ T cells in peripheral blood, spleen and lymph nodes (LNs) compared with control mice (Figure 3f). Similarly, in the spleens and lymph nodes, compared with control mice, HO‐*Dap5^ΔFoxp3^* mice showed a marked increase in Teff and CD8^+^ T (Figure S3g,h, Supporting Information).

We also observed markedly elevated concentrations of IL‐2, type I (IFN‐γ) and II cytokines (IL‐4, IL‐5, IL‐9 and IL‐13), and visibly increased type III cytokines levels (IL‐6, IL‐17A and IL‐17F) in the blood of HO‐*Dap5^ΔFoxp3^* (Figure S4a, Supporting Information). The HO‐*Dap5^ΔFoxp3^* mice also exhibited extremely high IgE concentrations in the plasma (Figure S4b, Supporting Information), indicating the occurrence of strong type‐II allergic reactions. The HO‐*Dap5^ΔFoxp3^* had markedly reduced B cells in peripheral blood and diminished BCR clonal expansion in comparison with control mice (Figure 3g,h), which were verified by flow cytometry detection in peripheral blood, spleen and LNs (Figure 3i; Figure S4c,d, Supporting Information). H&E and mIF results showed impaired germinal center (GC) structures in the spleens from HO‐*Dap5^ΔFoxp3^* mice (Figure 3j; Figure S4e, Supporting Information).

Severe Treg defects have been associated with immune‐related adverse events (irAEs), as evidenced in previous studies.^[^ ^28^, ^29^, ^30^, ^31^ ^]^ Consistently, HO‐*Dap5^ΔFoxp3^* mice developed severe systemic inflammation, indicating a breakdown of central immune tolerance resulting from compromised Treg function. To investigate the underlying causes, we detected alterations of Treg homeostasis in sick HO‐*Dap5^ΔFoxp3^* mice. Flow cytometry analysis demonstrated a significant reduction in Treg frequencies in multiple organs following conditional deletion of Dap5 in Tregs (Figure 3k; Figure S4f,g, Supporting Information). Besides, pTregs, rather than CD4^+^Tconv or CD8^+^ T cells, were largely apoptotic in 5 weeks old HO‐*Dap5^ΔFoxp3^* mice (Figure 3l).

We further performed adoptive T cell transfer experiment by intravenous injection (i.i.) of splenic naïve CD4^+^ T cells from HO‐*Dap5^ΔFoxp3^* or *Dap5^flox^* mice into *Rag1^−/−^* mice. *Rag1^−/−^* mice receiving *Dap5^ΔFoxp3^* naïve CD4^+^ T cells developed enlarged spleens and LNs and died ≈ 20 days post‐transfer (Figure 3m,n) and exhibited significantly reduced Treg abundance in the periphery (Figure S4j, Supporting Information). Taken together, these results demonstrate that Dap5 is indispensable for maintaining peripheral Treg survival and immunosuppressive function.

### 2.4 Tregs with Heterozygous Deletion of *Dap5* Displayed Intact Homeostasis in the Periphery

**Figure 4:**
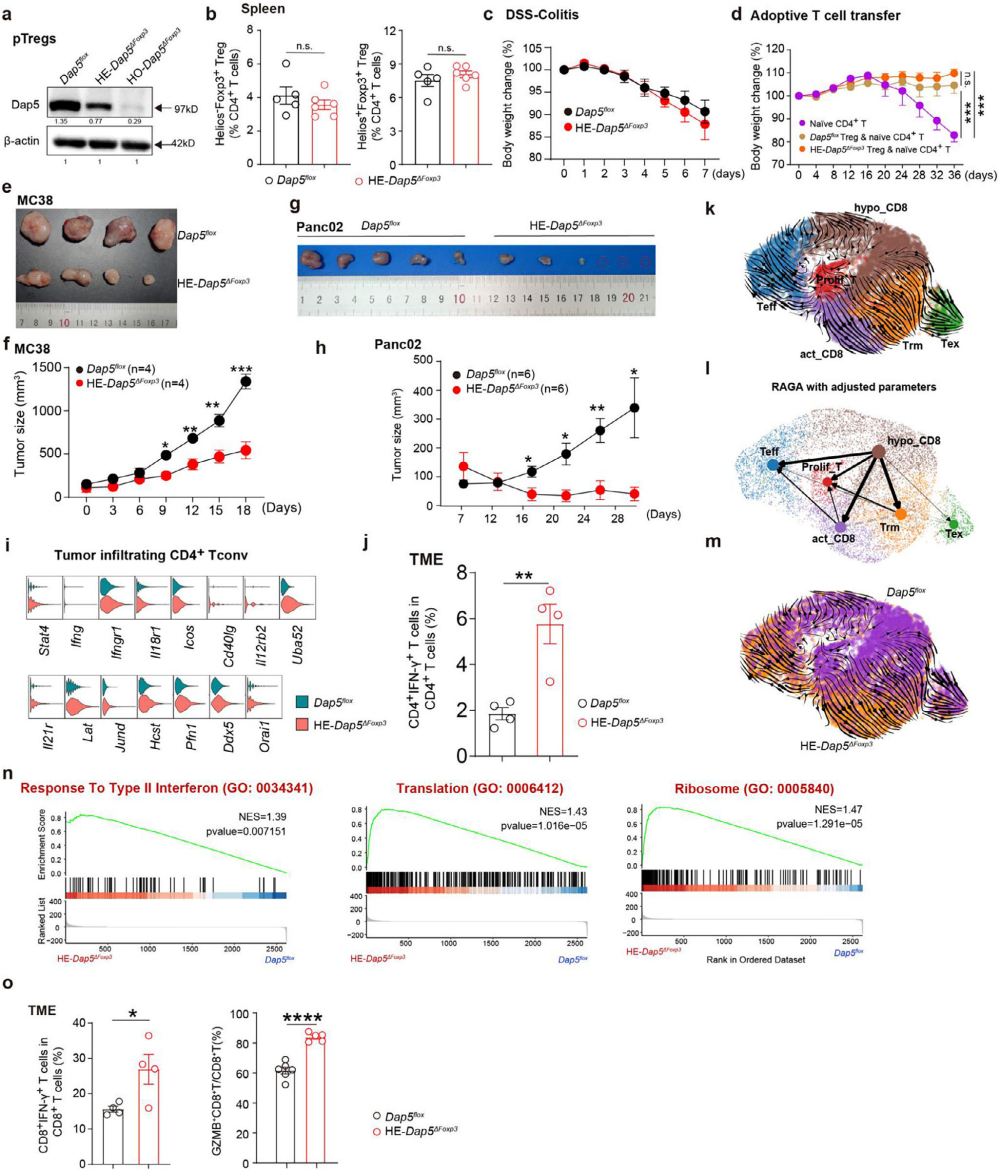
Mice with heterozygous Dap5 deletion in Tregs displayed unaltered peripheral immune homeostasis but enhanced Teff response against subcutaneous tumors. a) WB results showing intermediate expression level of Dap5 in pTregs from HE‐*Dap5^ΔFoxp3^* mice. b) Bar plot displaying unaltered splenic Treg frequencies in HE‐*Dap5^ΔFoxp3^* mice. c) Curves of body weight changes for HE‐*Dap5^ΔFoxp3^* and *Dap5^flox^* mice under DSS‐induced colitis modeling. d) Curves of body weight changes for *Rag1^−/−^* mice receiving adoptive transfer of naïve CD4^+^ T cells or naïve CD4^+^ T cells mixed with Tregs purified from HE‐*Dap5^ΔFoxp3^* mice or *Dap5^flox^* mice. e) Suppressed MC38 tumor growth in HE‐*Dap5^ΔFoxp3^* mice. f) Curves monitoring MC38 tumor growth in HE‐*Dap5^ΔFoxp3^* mice or *Dap5^flox^* mice. g) Suppressed Panc02 tumor growth in HE‐*Dap5^ΔFoxp3^* mice. h) Curves monitoring Panc02 tumor growth in HE‐*Dap5^ΔFoxp3^* mice or *Dap5^flox^* mice. i) Violin plots comparing gene expressions between tumor‐infiltrating CD4^+^ Tconv from HE‐*Dap5^ΔFoxp3^* and *Dap5^flox^* mice. j) Bar plots comparing infiltrations of IFN‐γ^+^CD4^+^ T cells in the MC38 tumors that grew in HE‐*Dap5^ΔFoxp3^* and *Dap5^flox^* mice. k) RNA velocity streamlines projected onto the UMAP‐based embedding. Cells were grouped according to their annotations. l) The directed Partition‐based Graph Abstraction (PAGA) graph showing the connectivity of these CD8^+^ T subpopulations. The edge weights quantify the connectivity between cell groups. m) Projection of RNA velocity streamlines on UMAP, grouped by cell type. n) GSEA plots displaying enriched signaling pathways in tumor‐infiltrating CD8^+^ T cells from HE‐*Dap5^ΔFoxp3^* mice in contrast to that from *Dap5^flox^* mice. o) Bar plots comparing infiltrations of IFN‐γ^+^CD8^+^ T and GZMB^+^CD8^+^ T cells between HE‐*Dap5^ΔFoxp3^* and *Dap5^flox^* mice. *p‐*values were determined by two‐tailed student's *T*‐test (b‐d, f, h, j and o), ^*^ *p*<0.05, ^**^ *p*<0.01, ^****^ *p*<0.001, ^***^ *p*<0.001.

The pTregs purified from HE‐*Dap5^ΔFoxp3^* mice showed intermediate Dap5 expression (**Figure** **4**a). The HE‐*Dap5^ΔFoxp3^* mice had unaltered Treg frequencies and did not spontaneously develop fatal autoimmune responses under steady state during the lifespan observed (until 12 months) (Figure 4b; Figure S5a, Supporting Information). H&E staining revealed no obvious inflammations in multi‐organs examined in HE‐*Dap5^ΔFoxp3^* mice (Figure S5b, Supporting Information). Moreover, the HE‐*Dap5^ΔFoxp3^* mice displayed marginally lighter (not statistically significant) body weight compared with the control *Dap5^flox^* mice when subjecting to dextran sulfate sodium (DSS)‐induced colitis modeling (Figure 4c; Figure S5c, Supporting Information). We next intravenously transferred naïve CD4^+^ T cells (from wild type C57BL/6J mice) mixed with Tregs purified from HE‐*Dap5^ΔFoxp3^* or *Dap5^flox^* mice into *Rag1^−/−^* mice (Figure S5d, Supporting Information) and found that Tregs from HE‐*Dap5^ΔFoxp3^* mice were equally capable of suppressing naïve CD4^+^ T‐induced colitis in *Rag1^−/−^* mice as Tregs from *Dap5^flox^* mice (Figure 4d; Figure S5e, Supporting Information). Considering that Dap5 deficiency in Tregs does not alter tTreg development and pTreg differentiation, we concluded that haploinsufficiency of Dap5 in Tregs does not affect their homeostasis and functionality under both steady state and acute inflammatory condition.

### 2.5 Haploinsufficiency of Dap5 in Tregs Leads to Enhanced Antitumor Teff Response

Wondering whether *Dap5* loss would affect Treg functionalities in TME, we observed subcutaneous tumor growth in HE‐*Dap5^ΔFoxp3^* mice. As expected, the growth of colorectal cancer cells (MC38) and pancreatic ductal adenocarcinomas (Panc02) were significantly suppressed in HE‐*Dap5^ΔFoxp3^* mice (Figure 4e–h). To delineate the alterations of immune microenvironment of TME in the HE‐*Dap5^ΔFoxp3^* mice, we performed scRNA‐seq with purified CD3^+^ T cells from subcutaneous tumors (Panc02). Besides T cells, we identified the presence of tumor cells (*Krt8* and *Krt18*), cancer associated fibroblasts (CAFs) (*Sparc* and *Dcn*), macrophages (Macs) (*Lyz2*) and mast cells (*Mcpt4* and *Gata2*) in the scRNA‐seq data due to possible non‐specific contaminations (Figure S5f,g, Supporting Information). Nevertheless, notably reduced tumor cells and CAFs were observed in samples from the HE‐*Dap5^ΔFoxp3^* group (Figure S5f, Supporting Information).

We further annotated ti‐Tregs (*Cd4* ^+^ *Foxp3^+^*), Tconv (*Cd4* ^+^ *Foxp3^−^*) and γδT17 (*Trdc* and *Il7a*) (Figure S5g, Supporting Information). Tconv from HE‐*Dap5^ΔFoxp3^* overexpressed genes involved in Th1 response (*Stat4*, *Ifng*, *Ifngr1*, *Il18r1*, *Cd40lg*, *Il12rb2*, *Il21r* and *Lat*) (Figure 4i), which were confirmed by the flow cytometric results showing increased IFN‐γ^+^CD4^+^ T cell infiltrations in tumors from HE‐*Dap5^ΔFoxp3^* mice (Figure 4j). ScRNA‐seq analysis annotated 6 subpopulations of tumor‐infiltrating CD8^+^ T cells (ti‐CD8^+^ T): activated CD8^+^ T (act _CD8) (*Lef1*, *Tcf7*, *Nfatc2*, *Rps3*, *Rpl8* and *Socs3*), proliferative CD8^+^ T (prolif_T) (*Mki67* and *Stmn1*), effector CD8^+^ T (Teff) (*Ifng*, *Il2rg*, *Gzmb*, *Isg15*, *Stat1*),^[^ ^32^ ^]^ tissue‐resident memory CD8^+^ T (Trm) (*Itgae/CD103*, *Lgals3*, *Itgb1*and *Itgb7*), exhausted CD8^+^ T (Tex) (*Lag3*, *Tox*, *Pdcd1* and *Havcr2*) and hyporesponsive CD8^+^ T (hypo_CD8) with non‐specific gene expression pattern (Figure S5h, Supporting Information). Results of RNA velocity analysis identified the multiple differentiation trajectories emanating from hypo_CD8 to act_CD8, Teff and Trm populations (Figure 4k,l), suggesting that hypo_CD8 are poorly activated and dysfunctional. Interestingly, ti‐CD8^+^ T from HE‐*Dap5^ΔFoxp3^* mice were polarized to Teff and Trm differentiation while the counterparts in *Dap5^flox^* were skewed toward the hypo_CD8 direction (Figure 4m), indicating that the TME in HE‐*Dap5^ΔFoxp3^* mice had promoted CD8^+^ T cell activation and differentiation.

Gene set enrichment analysis (GSEA) results revealed that ti‐CD8^+^ T from HE‐*Dap5^ΔFoxp3^* mice overexpressed genes enriched in the pathways of response to type II interferon (GO: 0034341, NES = 1.39, *p*‐value = 0.007151), ribosome (GO: 0005840, NES = 1.47, p‐value = 1.29 × 10^−5^) and translation (GO: 0006412, NES = 1.43, *p*‐value = 1.02 × 10^−5^) (Figure 4n). Flow cytometric results also showed increased presence of IFN‐γ^+^CD8^+^ T and GZMB^+^CD8^+^ T cells in tumors from HE‐*Dap5^ΔFoxp3^* mice (Figure 4o). Together, haploinsufficiency of Dap5 in Tregs results in enhanced Teff response in the TME.

### 2.6 DAP5 Undergo LLPS

**Figure 5:**
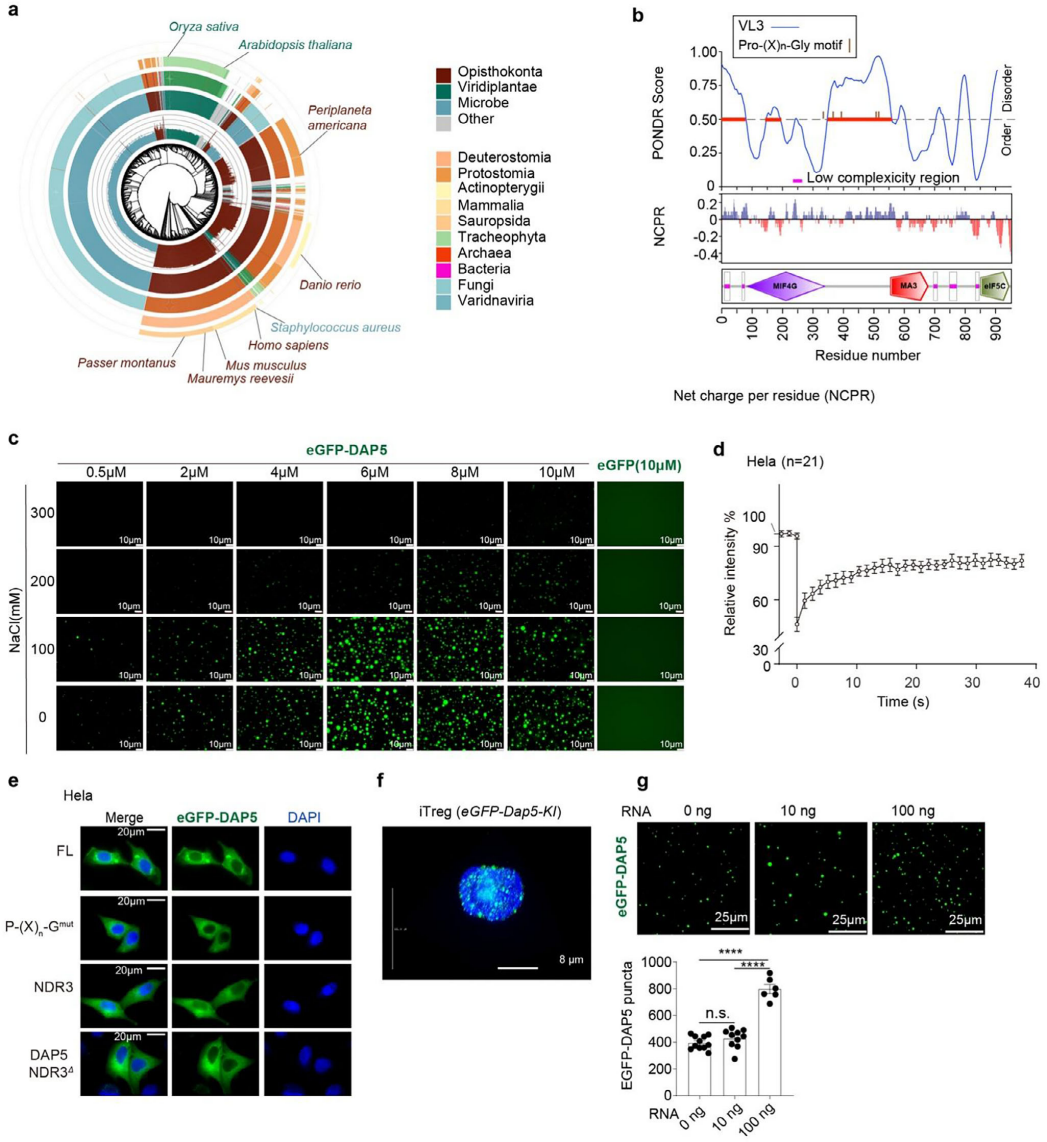
LLPS of DAP5. a) Phylogenetic tree plot showing that DAP5 is highly conserved in eukaryotic species. b) DAP5 domains aligned with the results of predictor of natural disordered regions (PONDR) and net charge per residue (NCPR) analyses. c) LLPS of purified recombinant eGFP‐DAP5 protein was observed in the buffer supplemented with serial concentrations of protein and NaCl under confocal microscopy. d) The fluorescence recovery after photobleaching (FRAP) curve illustrating the dynamic recovery of eGFP‐DAP5^+^ puncta in HeLa cells stimulated with 50 µM ETO for 12 h. e) HeLa cells were transfected with indicated DAP5 constructs, then exposed to 50 µM ETO for 12 h. Subcellular eGFP‐DAP5 distribution were subsequently visualized under fluorescence microscopy. f) Observation of eGFP‐Dap5^+^ puncta in iTregs generated from *eGFP‐Dap5‐KI* mice under confocal microscopy. g) Total RNAs purified from HeLa cells dose‐dependently enhanced the formation of eGFP‐DAP5^+^ puncta. *p‐*values were determined by two‐tailed student's *T*‐test (g), ^*^ *p*<0.05, ^**^ *p*<0.01, ^***^ *p*<0.001, ^****^ *p*<0.0001.

To elucidate the mechanistic basis of DAP5‐mediated acquired fitness of ti‐Tregs in TME, we performed in‐depth exploration of DAP5's molecular function. Phylogenetic analysis revealed that DAP5 is an evolutionarily conserved protein with prevalent presence among eukaryotes ranging from Fungi, Viridiplantae, Protostomia to Deuterostomia, whereas its occurrence in prokaryotes is scarce (**Figure** **5**a), suggesting that DAP5‐mediated AMT is evolutionarily fundamental for eukaryotes. RNA‐binding proteins (RBPs) and RNAs form membraneless organelles through LLPS in cells under stressful conditions.^[^ ^33^ ^]^ DAP5 is an RBP with three Naturally Disordered Regions^[^ ^34^ ^]^ (NDR1‐3) and the NDR3 contains 5 Pro‐(X)_n_‐Gly motifs^[^ ^35^ ^]^ (Figure 5b). AlphaFold prediction also showed a long NDR region between MIF4G and MA3 domain^[^ ^36^ ^]^ (Figure S6a, Supporting Information), suggesting that DAP5 is of LLPS potential. Indeed, recombinant DAP5 with eGFP tag at N‐terminus (eGFP‐DAP5) formed droplets in solution buffered with 200 mM Tris‐HCl (pH7.5), NaCl (0–100 mM) and 5% PEG8000, whereas eGFP failed in droplet formation. Higher DAP5 and lower NaCl concentration fortified DAP5‐droplet formation (Figure 5c). The DAP5 droplets underwent dynamic fusion process (Figure S6b, Supporting Information). We next expressed eGFP‐DAP5 protein in HeLa cells. The diffused cytosolic distribution of eGFP‐DAP5 in HeLa cells became condensed in puncta upon stimulation with the apoptosis inducer Etoposide (Eto, conc. 50 µM) or 42 °C heat shock for 10 min (Figure S6c, Supporting Information), photobleaching of the DAP5‐puncta recovered rapidly in ≈10 s (Figure 5d). DAP5 mutants with mutated Pro‐(X)_n_‐Gly motifs, NDR3 deletion (NDR3^Δ^) or NDR3 domain alone failed to undergo LLPS upon Eto induction in HeLa cells (Figure 5e). We generated an eGFP‐Dap5 knock‐in transgenic mouse strain (eGFP‐Dap5‐KI) (Figure S6d–f, Supporting Information). eGFP‐Dap5 in iTregs generated from eGFP‐Dap5‐KI mice was distributed in puncta (Figure 5f; Video S1, Supporting Information). Taken together, DAP5 molecules undergo LLPS in Tregs. Interestingly, total RNAs extracted from HeLa cells dose‐dependently promoted LLPS of DAP5 (Figure 5g).

### 2.7 DAP5 Bound to Pro‐Survival and Proliferative Transcripts with Active Translational Activities in Tregs

**Figure 6:**
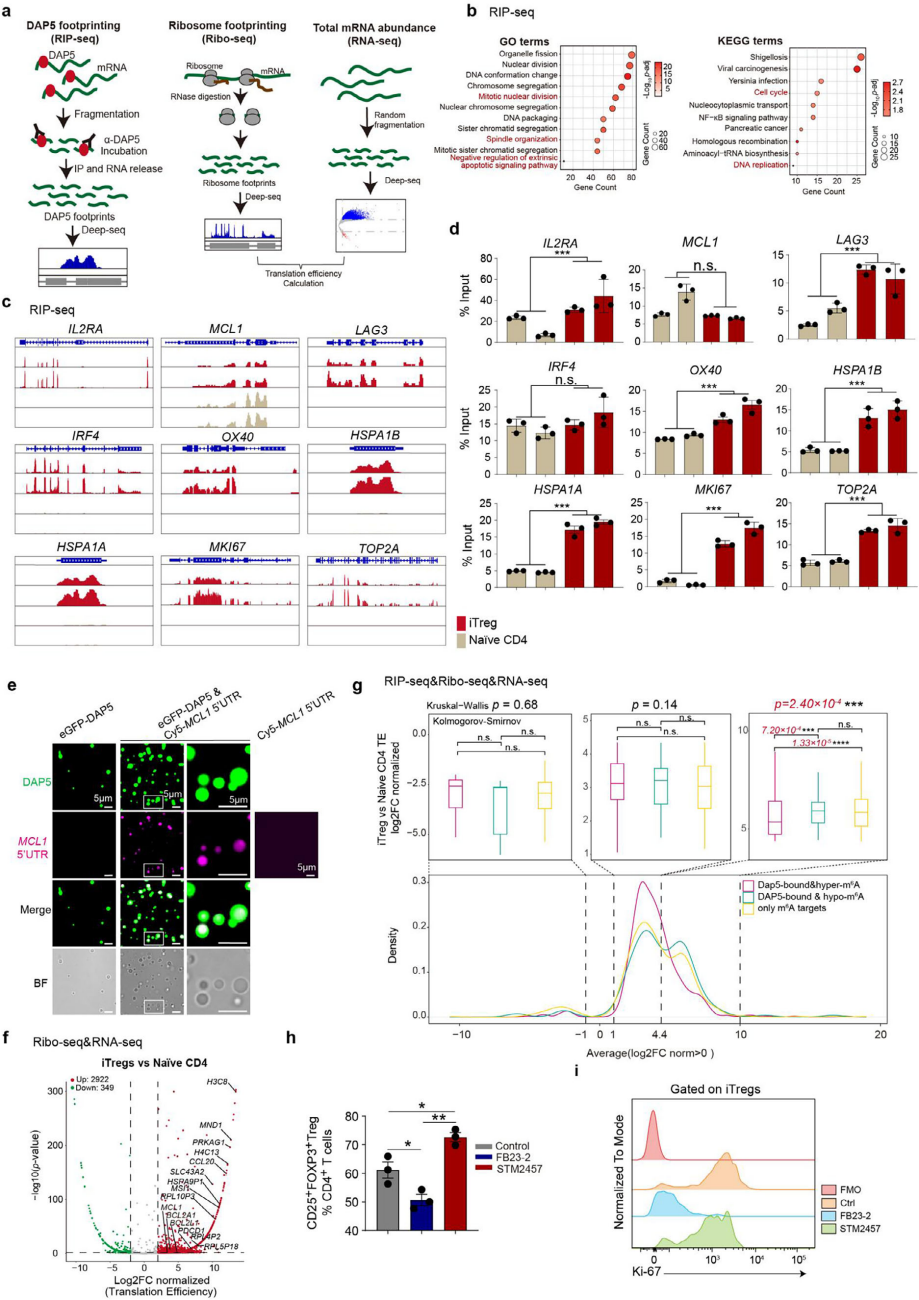
Transcripts bound by DAP5 undergo active translation in Tregs. a) Schematic diagram exhibiting the workflow for the combined RIP‐seq, Ribo‐seq and RNA‐seq experiments. b) Bubble plots displaying enriched GO and KEGG terms that were obtained by performing enrichment analysis with transcripts bound by DAP5 in iTregs. c) Integrative Genomics Viewer (IGV) tracks showing representative binding peaks of DAP5 that are associated with cell survival, proliferation and immunomodulation in human iTreg cells. d) Bar plots showing enriched presence of transcripts bound by DAP5 in iTregs. For histogram plotting, qRT‐PCR assays were performed with DAP5‐RIP assay elutes. The RIP‐qPCR data analysis was performed according to the ΔΔCt method.^[^ ^77^, ^78^ ^]^ Briefly, ΔCt [normalized RIP] = (Ct [RIP]‐(Ct [Input]‐Log_2_ (Input Dilution Factor))), % Input = 2^(‐ΔCt[normalized RIP])^. e) Addition of Cy5‐labeled 5′UTR of *MCL1* enhanced formation of eGFP‐DAP5^+^ puncta in the buffer containing 100 mM NaCl, 20 mM Tris‐HCl (pH = 7.5), 5% PEG8000 and 2 µM eGFP‐DAP5. Pictures were taken under confocal microscopy. f) The volcano plot depicting transcripts in iTregs with significantly increased translation efficiencies (TE) compared to naïve CD4^+^ T cells. TE values were determined by combined analysis of Ribo‐seq and RNA‐seq data. Transcripts meeting the criteria of absolute $\log_{2}\frac{\mathrm{TE}-\mathrm{iTreg}}{\mathrm{TE}-\mathrm{na}ï\mathrm{ve}\;\mathrm{CD}4}$ ≥ 2 and adjusted *p‐*value < 0.05 were designated as differentially translated. g) **Lower panel**: the curves displaying the density distributions of transcripts along their relative TE values ($\log_{2}\frac{\mathrm{TE}-\mathrm{iTreg}}{\mathrm{TE}-\mathrm{na}ï\mathrm{ve}\;\mathrm{CD}4})\;$(X‐axis). Transcripts were grouped into three categories: DAP5‐bound and hypo‐m^6^A modified, DAP5‐bound and m^6^A modified, and m^6^A modified only; **upper panel**: box plots comparing TE values of the aforementioned categories of transcripts within three indicated ranges of relative TE values: $\log_{2}\frac{\mathrm{TE}-\mathrm{iTreg}}{\mathrm{TE}-\mathrm{na}ï\mathrm{ve}\;\mathrm{CD}4}$<‐1, 1≤$\log_{2}\frac{\mathrm{TE}-\mathrm{iTreg}}{\mathrm{TE}-\mathrm{na}ï\mathrm{ve}\;\mathrm{CD}4}$<4.4, 4.4≤$\log_{2}\frac{\mathrm{TE}-\mathrm{iTreg}}{\mathrm{TE}-\mathrm{na}ï\mathrm{ve}\;\mathrm{CD}4}$≤10. h) Bar plots displaying efficiencies of in vitro mouse iTreg differentiation induced by FB23‐2 or STM2457. i) Flow cytometric histograms comparing Ki‐67 expression levels between mouse iTregs induced by FB23‐2 and STM2457. *P‐*values were determined by two‐tailed student's *T*‐test (d and h) or Kolmogorov‐Smirnov test and Kruskal‐Wallis (g), ^*^ *p*<0.05, ^**^ *p*<0.01, ^***^ *p*<0.001.

LLPS is attributed to intrinsically disordered regions (IDRs) of RBPs. Increasing evidence shows that these multivalent interactions between RNA molecules also contribute to form ribonucleoproteins with translational machinery to activate translation.^[^ ^37^, ^38^ ^]^ To globally map transcripts bound by DAP5 and simultaneously assess their translational activity in Tregs, we performed RNA immunoprecipitation (RIP‐seq) and sequencing using anti‐DAP5 antibody and ribosome sequencing (Ribo‐seq) coupled with RNA sequencing (RNA‐seq) with iTregs (**Figure** **6**a). With the same amount of input cells, more DAP5‐RIP‐seq peaks (n_1_ = 19718, n_2_ = 23030) were captured in iTregs than in naïve CD4^+^ T cells (n_1_ = 7788, n_2_ = 6587) (Figure S7a, Supporting Information). DAP5 in iTregs showed preferential bindings to coding sequence (CDS) versus introns (Figure S7b, Supporting Information). In general, there were 1023, 1706 and 1718 genes whose transcripts exhibited DAP5‐binding events specific to iTregs, naïve CD4^+^ T cells and both cell types, respectively (Figure S7c, Supporting Information). Transcripts bound by DAP5 in iTregs were involved in Treg stability, survival, and immunomodulatory function (*IL2RA*, *MCL1*, *LAG3*, *IRF4*, *OX40*), stress response (*HSPA1A*, *HSPA1B*), and proliferation (*MKI67*, *TOP24*) (Figure 6b,c). We validated the binding of DAP5 with these transcripts in iTregs by performing quantitative real‐time polymerase chain reaction (qRT‐PCR) with the DAP5‐RIP assay elutes (Figure 6d). In vitro and in vivo assay demonstrated that Cy5‐labeled 5′UTR of *MCL1* enhanced LLPS of DAP5 by incorporating into the DAP5^+^ puncta (Figure 6e; Figure S7d, Supporting Information). We further compared ribosome footprint profiles between human iTreg and naïve CD4^+^ T cells. The results of the P‐site analysis revealed that ribosome‐protected fragments (RPFs) in iTregs were being translated more actively compared to naïve CD4^+^ T cells (Figure S7e, Supporting Information). Coupled analysis of RNA‐seq and Ribo‐seq revealed that the pro‐proliferative and pro‐survival transcripts had higher translation efficiency in iTregs (Figure 6f). We cloned 5′Un‐Translational Regions (5′UTR) of *MKI67*, *MCL1* and *BCL2* into the bicistronic plasmid respectively, generating single transcript, in which translation of renilla luciferase (RLuc) was directed in CDT mode whereas translation of firefly luciferase (FLuc) was driven by 5′UTR‐mediated alternate mode. Results of the dual‐Luc assay showed that these 5′UTRs were capable of driving the production of FLuc in alternate mode and DAP5 overexpression markedly potentiated these effects (Figure S7f, Supporting Information).

N^6^‐Methyladenosine (m^6^A) is the most prevalent internal modification found in eukaryotic mRNAs, regulating mRNA metabolism and translation,^[^ ^39^ ^]^ we further profiled the m^6^A landscape of iTregs by performing MeSeq. We found that DAP5 bound to both hyper‐ and hypo‐m^6^A modified transcripts in iTregs, however, larger portion of DAP5‐bound‐hypo‐m^6^A transcripts exhibited high translational efficiency (TE) when compared with DAP5‐bound‐hyper‐m^6^A transcripts in iTregs (Figure 6g). Addition of the m^6^A writer inhibitor (10 µM STM2457) significantly promoted mouse iTreg differentiation, whereas administration of the m^6^A eraser inhibitor (10 µM FB23‐2) substantially suppressed mouse iTreg differentiation (Figure 6h,i), suggesting that Dap5 may preferentially initiate the translation of transcripts with hypo‐m^6^A modification levels.

### 2.8 Dap5 Drives the Translation of CD25 and MCL‐1 to Facilitate Treg Stability and Survival in TME

**Figure 7:**
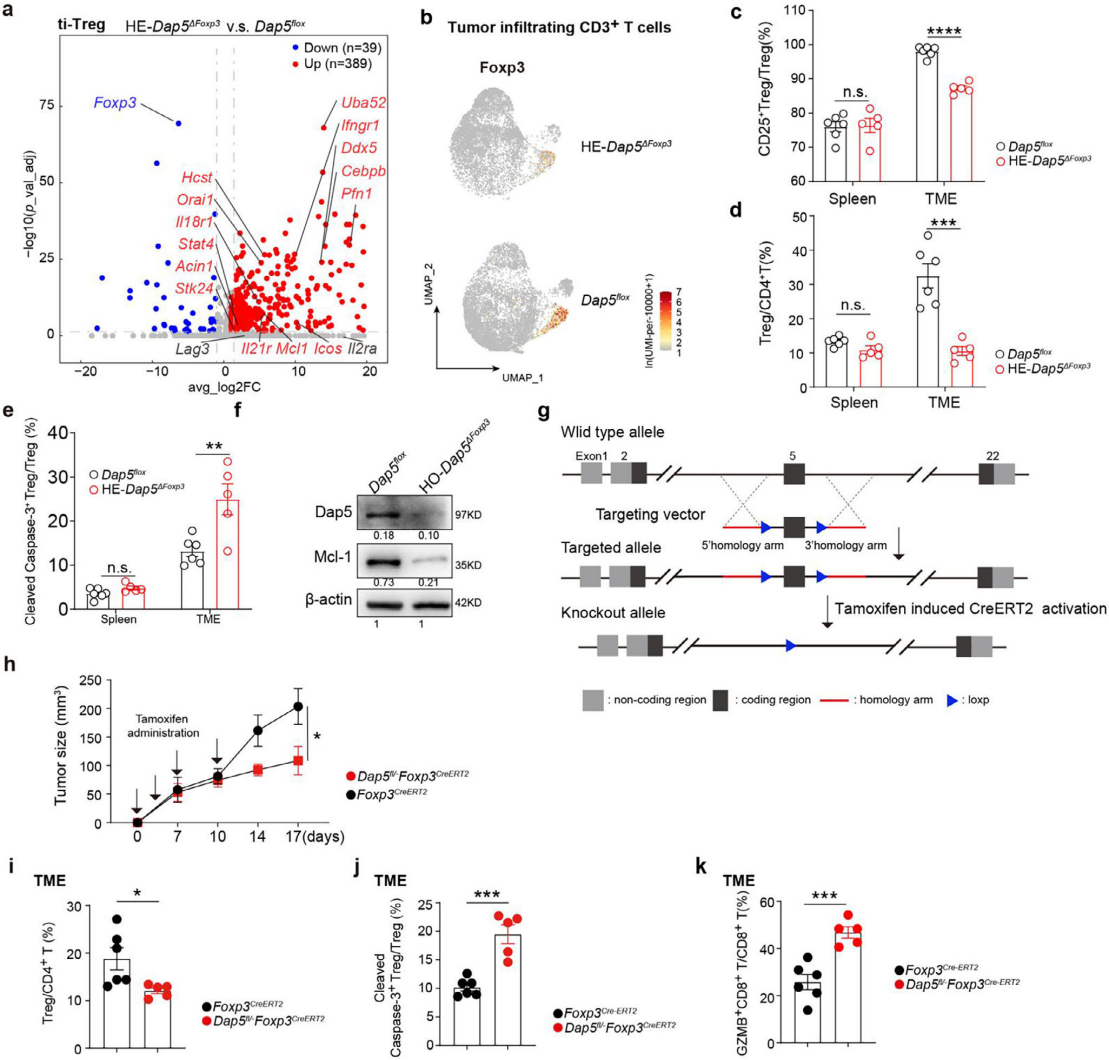
Dap5 promotes *IL2RA* and *MCL1* translation to maintain ti‐Treg stability and survival. a) Volcano plots showing differentially expressed genes between ti‐Tregs from HE‐*Dap5^ΔFoxp3^* or *Dap5^flox^* mice. b) 2D‐UMAP plots displaying reduced *Foxp3* expression among ti‐Tregs from HE*‐Dap5^ΔFoxp3^* mice. c) Bar plots comparing CD25 expressions in splenic or tumor‐infiltrating CD4^+^Foxp3^+^T cells between HE‐*Dap5^ΔFoxp3^* or *Dap5^flox^* mice. d) Bar plots comparing frequencies of splenic or tumor‐infiltrating CD4^+^Foxp3^+^ T cells between HE‐*Dap5^ΔFoxp3^* or *Dap5^flox^* mice. e) Bar plots comparing proportions of splenic or tumor‐infiltrating cleaved‐caspase3^+^ Tregs between HE‐*Dap5^ΔFoxp3^* or *Dap5^flox^* mice. f) Immunoblotting results showing absence of Mcl‐1 expression in iTregs derived from HO‐*Dap5^ΔFoxp3^*. g) Construction strategy for *Dap5^flox^Foxp3^CreERT2^* strain. h) Curves monitoring MC38 tumor growth in *Dap5^fl/+^Foxp3^CreERT2^* and *Foxp3^CreERT2^* mice. Tamoxifen was administered on the same day as tumor cell inoculation. i‐k) Bar plots comparing proportions of Tregs in CD4^+^T cells (i), cleaved caspase‐3^+^ Tregs in total Tregs (j) and GZMB^+^CD8^+^ T in total CD8^+^ T cells (k) in tumors from *Dap5^fl/+^Foxp3^CreERT2^* and *Foxp3^CreERT2^* mice. *P‐*values were determined by two‐tailed student's *T*‐test (c, f, h, i, j and k) ^*^ *p*<0.05, ^**^ *p*<0.01, ^***^ *p*<0.001, ^****^ *p*<0.0001.

Since RIP‐seq and functional assays indicated that Dap5 preferentially engages pro‐survival and stability‐associated transcripts in Tregs, we next examined whether Dap5 regulates their translation to support ti‐Treg stability and survival in TME. Transcriptomic comparison of ti‐Tregs between HE‐*Dap5^ΔFoxp3^* and *Dap5^flox^* mice obtained 39 downregulated genes and 389 upregulated genes (absolute Log2FC value >1, *p*_val_adj<0.05) (**Figure** **7**a). Astonishingly, ti‐Tregs from HE‐*Dap5^ΔFoxp3^* mice displayed substantially decreased *Foxp3* expression but increased expressions of genes associated with Th1 signature (*Stat4*, *Ifngr1*, *Il18r1*, *Hcst*, *Orail1* and *Icos*) and cell death (*Stk24* and *Acin1*) (Figure 7a,b), suggesting that HE‐*Dap5^ΔFoxp3^* ti‐Tregs were defective in lineage stability maintaining and prone to Th1 polarization and apoptosis. For mature Tregs, the IL‐2/CD25 signaling pathway plays pivotal roles to sustain their stability, survival and proliferation.^[^ ^40^, ^41^, ^42^ ^]^ The flow cytometric results showed that in HE‐*Dap5^ΔFoxp3^* mice, CD25 expression on Tregs were significantly decreased in TME but remained unchanged in the spleens (Figure 7c). Given the fact that DAP5 physically binds to *IL2RA* and *MCL1* transcripts in Tregs (Figure 6c,d) and *Il2ra* expression in ti‐Tregs was unchanged between HE‐*Dap5^ΔFoxp3^* and *Dap5^flox^* group (Figure 7a), we believed that CD25 expression was impaired at translation level in ti‐Tregs from HE‐*Dap5^ΔFoxp3^* mice.

Treg frequencies were significantly reduced in the TME of HE‐*Dap5^ΔFoxp3^* mice, but not in the spleens (Figure 7d). Consistently, an increased proportion of cleaved Caspase‐3^+^ ti‐Tregs was observed in HE‐*Dap5^ΔFoxp3^* mice (Figure 7e). Given that Mcl‐1 is a key anti‐apoptotic factor required for Treg survival^[^ ^43^ ^]^ and Dap5 physically binds *Mcl1* transcripts in Tregs (Figure 6c,d), we assessed Mcl‐1 protein expression. iTregs from *Dap5^ΔFoxp3^* mice showed markedly decreased Mcl‐1 protein levels (Figure 7f). Interestingly, *Mcl1* transcript levels were upregulated in ti‐Tregs from HE‐*Dap5^ΔFoxp3^* mice (Figure 7a). Together, these findings indicate that Dap5 promotes Mcl‐1 expression at translation level, thereby supporting Treg survival in the TME.

To explore the potential of targeting Dap5 for the treatment of established tumors, we generated *Dap5^fl/+^Foxp3^CreERT2^* mice to partially delete Dap5 in Tregs upon tamoxifen treatment when the tumor was established (Figure 7g). Tumor growth was significantly reduced in *Dap5^fl/+^Foxp3^CreERT2^* mice (Figure 7h). Flow cytometric results revealed that tumors from *Dap5^fl/+^Foxp3^CreERT2^* mice exhibited a significant reduction in ti‐Treg infiltration (Figure 7i), accompanied by increased ti‐Treg apoptosis (Figure 7j) and enhanced infiltrations of GZMB^+^CD8^+^ T cells (Figure 7k).

## 3 Discussion

**Figure 8:**
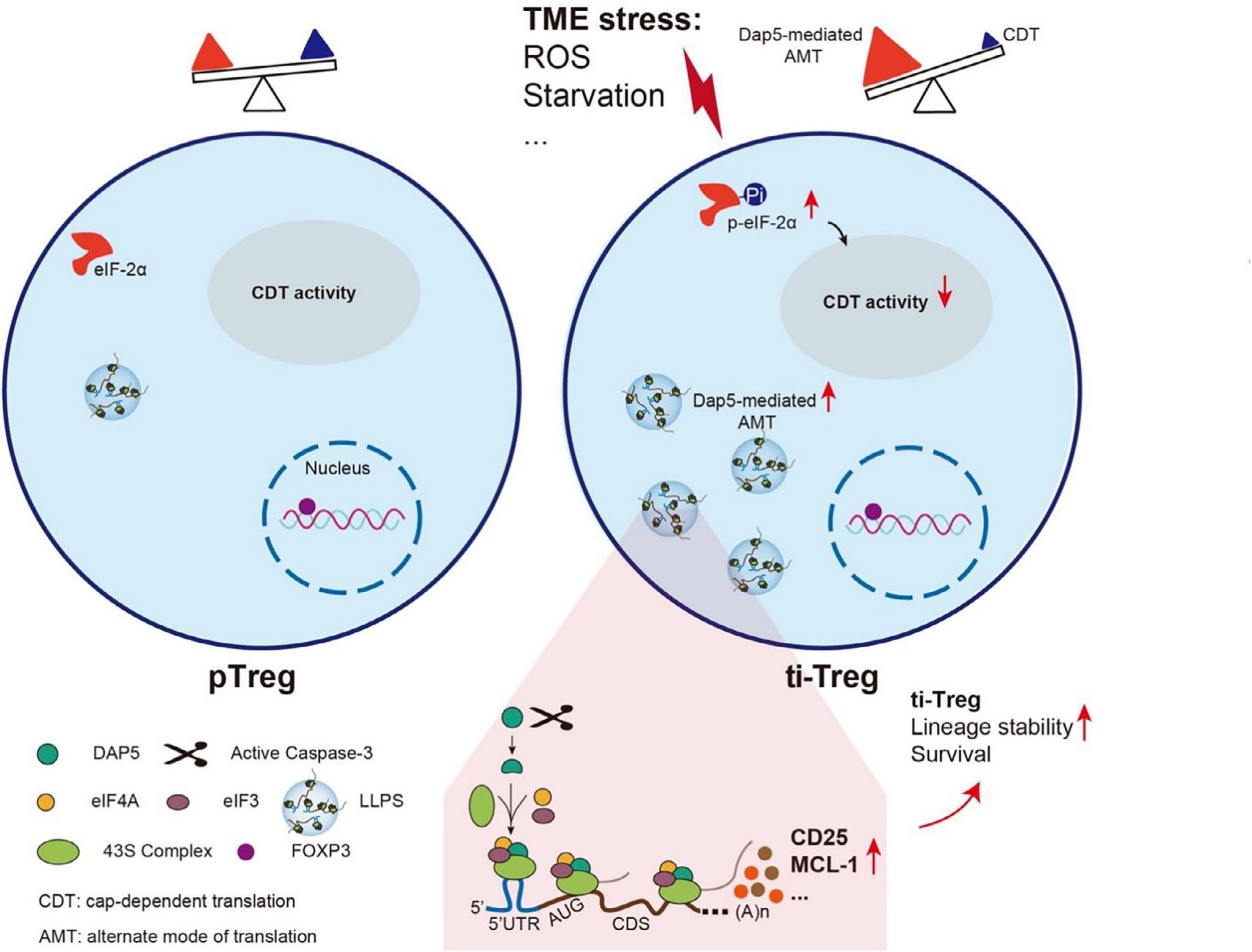
Graphical abstract: Dap5 functions as a molecular switch of translation mode in ti‐Tregs. Chronic stresses in the TME trigger ISR, leading to eIF‐2a phosphorylation and a consequent impairment of CDT activity in Tregs. Meanwhile, ti‐Tregs engage Dap5 to sustain alternate mode of translation of CD25 and MCL‐1, which are critical for ti‐Treg lineage stability and survival in the harsh TME.

The metabolically and immunologically stressful TME creates an imbalance by suppressing antitumor Teff while concurrently promoting the fitness and stability of Tregs.^[^ ^7^, ^9^, ^10^, ^11^, ^12^, ^13^, ^43^, ^44^, ^45^ ^]^ In this study, we identify Dap5 as a key determinant of acquired fitness of Treg under stressful TME. Using genetic ablation models, we show that Dap5 is dispensable for thymic Treg development but essential for the homeostasis and function of peripheral Tregs. Importantly, partial loss of Dap5 selectively impaired ti‐Treg stability while sparing peripheral immune tolerance, thereby unleashing antitumor immunity. Mechanistic studies revealed that Dap5 functions as a molecular switch of translation in Tregs (**Figure** **8**). While canonical CDT activity is attenuated, Tregs engage Dap5 to sustain alternate mode of translation (AMT) of key proteins, including CD25 and MCL‐1, which are critical for ti‐Treg lineage stability and survival. This context‐dependent role of Dap5 distinguishes Tregs from Teff and highlights Dap5‐mediated AMT as a central mechanism shaping ti‐Treg fitness in the TME.

The HO‐*Dap5^ΔFoxp3^* mice spontaneously developed lethal autoimmune responses. Further clarification revealed that HO‐*Dap5^ΔFoxp3^* mice exhibited hyper‐activated type I and II immune responses, slightly enhanced type III response but substantially diminished GC B cell response. We believe that under steady state, Tregs with *Dap5* loss are incapable of consuming peripheral IL‐2, making mutant mice predisposed to systemic autoimmune responses due to accumulated IL‐2 amount, as high level of IL‐2 promotes cytotoxic CD8^+^ T, Th1 and Th2 differentiation but suppresses Th17 and Tfh differentiation and GC response.^[^ ^46^, ^47^, ^48^, ^49^ ^]^

Ti‐Tregs are key drivers of the immunosuppressive TME and are associated with poor prognosis across multiple cancer types.^[^ ^2^ ^]^ We show that DAP5 expression is specially elevated in ti‐Tregs, correlating with effector Treg signature in human colorectal tumors. Strikingly, Dap5 haploinsufficiency selectively impaired Ti‐Treg function and suppressed tumor growth without compromising systemic tolerance. Given prior evidence that Dap5 acts as an oncogenic driver in multiple cancers‐including hepatocellular carcinoma, diffuse large B‐cell lymphoma, acute myeloid leukemia, glioblastoma, and gastric cancer^[^ ^50^, ^51^, ^52^, ^53^, ^54^ ^]^‐pharmacologic targeting of Dap5 may achieve dual benefits: directly impairing tumor growth while simultaneously reducing ti‐Treg mediated immunosuppression and enhancing effector T cell responses. However, complete ablation of Dap5 in Tregs caused lethal systemic autoimmunity, emphasizing the necessity of tumor‐localized or partial inhibition strategies to minimize collateral immune dysregulation.

DAP5 undergoes liquid‐liquid phase separation (LLPS) in response to stress, forming dynamic condensates that are promoted by mRNAs such as *MCL1* 5′UTR. This suggests that DAP5 may organize specialized translational hubs to facilitate selective protein synthesis under conditions when CDT is suppressed. While LLPS of RNA‐binding proteins has been implicated in diverse processes including antiviral responses, circadian translation, and spermatogenesis,^[^ ^37^, ^55^, ^56^, ^57^ ^]^ our study provides the first evidence that DAP5 condensation may underpin alternative translation in Tregs. Future studies are required to determine whether LLPS constitutes the structural basis for selective mRNA translation in immune regulation.

m^6^A enhances the phase separation potential of mRNA, influencing mRNA folding, stability, degradation and translation,^[^ ^39^, ^58^ ^]^ which plays significant roles in the differentiation and functional regulation of immune cells including dendritic cells, macrophages and T cells.^[^ ^59^, ^60^, ^61^ ^]^ Previous reports have shown that deletion of m^6^A reader *Ythdf2* in Tregs affected the survival and immunosuppressive function of Tregs in TME while leaving peripheral immune homeostasis intact,^[^ ^62^ ^]^ and mice with *Mettl3* ablation in Tregs developed spontaneous lethal autoimmune response.^[^ ^61^ ^]^ However, here we found that the hypo‐m^6^A modified transcripts bound by DAP5 displayed higher translational efficiencies and administration of m^6^A eraser inhibitor suppressed iTreg differentiation. The detailed mechanisms responsible for the homeostasis and functionality of Tregs in both periphery and TME may be context‐dependent and warrant further investigations.

Although RIP‐seq identified a broad repertoire of DAP5‐associated transcripts, in vivo validation revealed MCL‐1 and CD25 (encoded by *IL2RA*) as the most functionally relevant targets. Reduced expression of MCL‐1 explained the increased apoptosis of Dap5‐deficient Tregs,^[^ ^43^, ^63^ ^]^ while diminished expression of CD25 on Tregs compromises their ability to capture IL‐2, thereby impairing their suppressive function.^[^ ^64^, ^65^ ^]^ These two nodes provide a mechanistic link between DAP5‐dependent translation and the dual hallmarks of Treg biology: survival and suppressive capacity.

Importantly, our findings suggest potential translational applications in cancer immunotherapy. Immune checkpoint blockade (ICB) therapies targeting PD‐1, PD‐L1, or CTLA‐4 have revolutionized cancer treatment by enhancing effector T cell responses, yet these therapies can paradoxically expand immunosuppressive ti‐Tregs, thereby limiting efficacy.^[^ ^3^, ^66^ ^]^ By selectively targeting DAP5‐mediated translation in ti‐Tregs, it may be possible to destabilize these suppressive cells within the TME without compromising systemic immune tolerance. Such an approach could synergize with existing ICB therapies by simultaneously enhancing Teff activity and reducing ti‐Treg‐mediated immunosuppression. Moreover, pharmacologic DAP5 inhibitors could offer a dual benefit: directly impairing tumor cell proliferation (as DAP5 is also oncogenic in multiple cancers) and reprogramming the immune landscape to favor antitumor responses. This strategy warrants further preclinical investigation to optimize dosing and delivery, ensuring maximal tumor‐specific efficacy while minimizing the risk of systemic autoimmunity.

## 4 Experimental Section

### Ethical Statement and Study Approval

The acquisition and use of human specimens were in compliance with the International Ethical Guidelines for Research Involving Human Subjects as stated in the Helsinki Declaration and were approved by the Ethics Committee at Shanghai General Hospital (No.:2024KS269). All the tissue samples were obtained upon signed informed consent from the participants. All the animal operations were approved by Shanghai General Hospital and in compliance with ethical regulations for animal research (IACUC: 2023AW053). The pathological examinations for each specimen in the human CRC cohort were performed by two professional pathologists.

### Mice

*Foxp3*‐*Cre* (B6.129(Cg)‐*Foxp3^4(YFP/icre)Ayr^*/J, Cat#01 6959), *Foxp3^eGFP‐Cre‐ERT2^* (*Foxp3^9(EGFP/cre/ERT2)Ayr^*/J, cat#01 6961), *Rag1^−/−^* (B6.129S7‐*Rag1^1Mom^*/J, Cat#002216), CD45.1 (B6.SJL‐*Ptprc^a^Pepc^b^*/BoyJ, Cat#002014) were originally obtained from Jackson Laboratory. *Dap5^flox^* and *EGFP*‐*Dap5*‐*KI* mice under the background of C57BL/6J were created at Shanghai Model Organisms Center, Inc. All the mouse strains used in this project were bred and maintained in a specific pathogen‐free (SPF) condition at Shanghai General Hospital with a standard condition (12 h cycle of light and darkness, 20–24 °C, 40–60% humidity). The gender of mice has no effect on the experiment, and both male and female mice at 6‐16 weeks of age were used for experiment.

### DNA Constructs

To generate the *EGFP‐DAP5* plasmid, we inserted the cDNA of human DAP5 into the pEGFP‐C1 vector. Considering that pro‐DAP5 would be cleaved by caspase‐3 at the position of 790 and turn into mature form P86,^[^ ^67^ ^]^ we chose to place EGFP at the N‐terminus of DAP5 and inserted 4×GS linker between the EGFP and DAP5 sequences, resulting in the fusion expression of EGFP‐linker‐DAP5 protein. The cDNA sequences of P‐(X_n_)‐G,^mut[^ ^35^ ^]^ NDR and DAP5^△NDR3^ were inserted into the pEGFP‐C1 vector as the same way. To construct the Dual Luciferase Reporter, it generated the 5′ UTR sequences of *MCL1*, *BCL2*, or *MKI67* mRNAs and linked them with the Rluc sequence at the N‐terminal along with the Fluc sequence at the C‐terminal.

### Cell cultures and Transfection

The HeLa (RRID: CVCL_0030, ATCC Cat# CCL‐2), MC38 (RRID: CVCL_B288, Cytion Cat# 305223), and Panc02 (RRID: CVCL_D627, Cytion Cat# 300501) cell lines were purchased from the indicated suppliers in 2020, 2022, and 2022, respectively. All cell lines were authenticated by short tandem repeat (STR) profiling and tested negative for mycoplasma contamination prior to use. The HeLa, MC38 and Panc02 cells were maintained in DMEM (Gibco, Cat#C11995500BT) supplemented with 10% FBS (Gibco, Cat#16000‐044) and 1% Penicillin‐Streptomycin solution (Gibco, Cat#15140122) and cultured at 37 °C in 5% CO_2_ air atmosphere. The *EGFP‐DAP5* plasmid was transiently transfected into HeLa cells using Lipofectamine 2000 (Invitrogen, Cat#11668019) following the manufacturer's instructions. Subsequently, the Cy5 labeled *MCL1* 5′UTR were transiently transfected into HeLa‐*EGFP‐DAP5* cells using RNAiMAX (Invitrogen, Cat#13 778 150). The Panc02 cells were cultured in RPMI‐1640 medium (Gibco, Cat#C11875500B) supplemented with 10% FBS and 1% Penicillin‐Streptomycin solution at 37 °C in 5% CO_2_ air atmosphere.

### Primary T Cell Isolation and In Vitro T Cell Differentiation

Mouse naïve CD4^+^ T cells were isolated from spleen of mice by flow cytometric sorting based on their surface marker (CD4^+^CD25^−^CD44^−^CD62L^+^). The purified naïve CD4^+^ T cells were maintained in RPMI 1640 supplemented 10% FBS and 1% penicillin‐streptomycin. Subsequently, mouse Th1, Th2, Th17 and iTreg differentiation were performed using the CellXVivo Mouse Th1 Cell Differentiation Kit (R&D Systems, Cat#CDK018), CellXVivo Mouse Th2 Cell Differentiation Kit (R&D Systems, Cat#CDK019), CellXVivo Mouse Th17 Cell Differentiation Kit (R&D Systems, Cat#CDK017), and CellXVivo Mouse Treg Cell Differentiation Kit (R&D Systems, Cat#CDK007), respectively, following the manufacturer's instructions. The differentiated cells were then analyzed using a flow cytometer (BD FACSCanto System) for further characterization and quantification of the differentiated cell populations. Human naïve CD4^+^ T cells were purified using the EasySep Human Naïve CD4^+^ T Cell Isolation Kit (Stemcell, Cat#19555). Subsequently, human iTreg differentiation was induced using a combination of human TGF‐β1 (2 ng mL^−1^, PeproTech), hIL‐2 (30U mL^−1^, PeproTech, Cat#200‐02), anti‐CD3/anti‐CD28 coated beads (Gibco, Cat#100‐21C), 0.05 mM β‐mercaptoethanol (ThermoFisher, Cat#21985023), and 20 nM Everolimus (mTORC1 inhibitor, MCE, Cat# HY‐10218). After culturing for 4 days, the cells were utilized for downstream experiments.

### Immunofluorescence and Microscopy

For cell imaging, the HeLa‐EGFP‐DAP5‐Cy5 *MCL1* mRNA 5′UTR cells were cultured in the 8‐well Nunc Lab‐Tek Chamber Slides (Thermo Scientific, Cat#177402) or 24‐well Round Coverslip (Solarbio, Cat#YA0350), and treated with 50 µM ETO (Sigma‐Aldrich, Cat#SRP2017) or subjected to heat shock at 42 °C for 10 min. For each of the fresh CRC specimens, the cancerous and paracancerous region were dissected and then fixed and embedded in paraffin. These formalin‐fixed and paraffin‐embedded (FFPE) samples were subsequently prepared into a tissue microarray for mIF staining. For splenic or FFPE section staining, PANO Multiplex IHC kit (Panovue) was used to examine the expressions of a panel of markers using antibodies of anti‐CD3 (Abcam, Cat#ab135372), anti‐CD19 (CST, Cat#90176T), anti‐CD4 (PathAb, Cat#PA448), anti‐FOXP3 (PathAb, Cat#PA285) and anti‐DAP5 (Santa Cruz, Cat#sc‐374236). In brief, slides were permeabilizated with 0.1% Triton X‐100 (Beyotime Cat#P0096) for 10 min followed by three washes with TBST and blocking with 10% goat serum (Beyotime, Cat#C0265) for 1 h at RT. After the first primary antibody was applied, the slides were incubated with secondary antibodies, followed by fluorochrome staining (fluorochrome: tyramide = 1:200) to amplify the signal. Subsequently, the slides were subjected to antigen retrieval in EDTA buffer (Beyotime, Cat#P0084) and were blocked with 10% goat serum. The immune‐hybridization process was then repeated for the remaining antigens. Once all antigens were labeled, the nuclei were stained with DAPI (Servicebio, Cat#G1012). The stained slides were scanned with the slide scanner (TissueGnostics, TissueFAXS Spectra S).

### Fluorescence Recovery After Photobleaching (FRAP)

The HeLa cells expressing eGFP‐DAP5 cultured in the 8‐well chamber slides were treated with 50 µM Eto for 12 h. FRAP were conducted on an inverted lase‐scanning confocal microscope equipped with a 63×oil immersion objective. Rectangular area that contained eGFP‐DAP5 foci was considered as the bleaching area. The selected areas were photobleached using the maximum intensity of 488‐nm imaging laser. FRAP was monitored at minimum intervals (≈2 s).

### In Vitro Transcription

In vitro transcription was performed using the mRNA Synthesis Kit (APEXBIO, Cat#E2040S) following the manufacturer's instructions. In brief, DNA plasmid containing *MCL1* 5′UTR were linearized. The T7 promoter sequence was added to the 5′ terminus of the DNA template (*MCL1* 5′UTR) by performing PCR. The DNA templates (5′‐T7 promoter‐*MCL1* 5′UTR‐3′) were amplified and purified. To create Cy5 labeled *MCL1* 5′UTR mRNA, in vitro transcription was carried out in the reaction mixture (20 µL) containing 1 µg of the DNA templates (5′‐T7 promoter‐*MCL1* 5′UTR‐3′), 2 mM ATP, 2 mM GTP, 2 mM CTP, 1.5 mM UTP, 0.5 mM Cy5‐UTP and 2 µL of T7 RNA Polymerase Mix at 37 °C for 4 h. The template DNA was degraded by DNase I at 37 °C for 15 min to stop the transcription. RNAs were purified using the RNA Clean and Concentrator Kit (APEXBIO, Cat#K1069) following the manufacturer's instructions.

### Recombinant Protein Purification

The pET‐28 vector harboring the construct of 6×His‐*EGFP‐DAP5* were transformed into DE3 *E. coli* cells and cultured overnight at 37 °C in solid agar plates. Single clone of *E. coli* was selected and cultured in LB medium at 37 °C until the OD600 reached 0.6–0.8. IPTG was then added to a final concentration of 0.5 mM to induce protein expression at 15 °C for 16 h. *E. coli* cells were centrifuged and the pelleted were lysed by sonication in lysis buffer accompanied with Protease Inhibitor (Beyotime, Cat#P1025). Ni‐affinity chromatography (Beyotime, Cat#P2241) was performed to purify the recombinant protein. The equilibration buffer was 50 mM Tris pH 8.0, 10% Glycerol. The wash buffer was 50 mM Tris, 10% Glycerol, pH 8.0 with 50 mM imidazole. The elution buffer was 50 mM Tris, 10% Glycerol, pH 8.0 with 500 mM imidazole. The eluted sample was further purified by ion exchange chromatography (Q‐HP). The eluted sample was dialyzed against a buffer containing 20 mM Tris, 1 mM DTT, pH 7.0. The final purified recombinant protein was freeze‐dried and stored at −80 °C.

### Immunoblotting

The cell lysates or mouse tissue lysates were prepared with RIPA buffer (Beyotime, Cat#P0013B) containing proteinase inhibitor cocktail (Beyotime, Cat#P1011) following the manufacturer's instructions. The lysates were then centrifuged at 4 °C for 10 min at 12000 g. The supernatant was mixed with 5× Protein Sample Loading Buffer (Epizyme, Cat#LT101) and boiled at 95 °C for 5 min. Subsequently, the samples were separated on 6%‐12% PAGE Bis‐Tris gels (Epizyme, Cat# LT102) and then transferred onto Immun‐Blot PVDF Membranes (Bio‐Rad, Cat#1620177). The membranes were blocked with QuickBlock buffer (Beyotime, Cat#P0252) and incubated with primary antibodies (anti‐β‐actin antibody, abcam, Cat#ab49900, 1:1000; anti‐MCL‐1 antibody, Abcam, Cat#32087, 1:1000; anti‐DAP5, Santa Cruz, Cat#sc‐37423, 1:1000) overnight at 4 °C. After primary antibody incubation, the membranes were washed three times with TBST and then incubated with HRP‐labeled Goat Anti‐Mouse/Rabbit IgG(H+L) (abcam, cat#ab6721 and cat#ab6789) for 1 h at RT. Following the secondary antibody incubation, the membranes were washed again three times with TBST and developed using an ECL chemiluminescence detection kit (Amersham, Cat# RPN2232). The signals were captured on X‐ray film (Tanon) for visualization.

### Dextran Sulfate Sodium (DSS) Induced Colitis Model

DSS (MP Biomedicals, Cat#0216011090) was dissolved in drinking water to the concentration of 2%. Mice were given access to the DSS solution for 7 days. Throughout the DSS treatment period, mice were monitored for signs of colitis, such as weight loss, diarrhea, rectal bleeding, and changes in stool consistency. At the end of the treatment period, mice were euthanized, and the colons were collected for evaluation of inflammation severity and histopathological analysis.

### Bone Marrow Chimerism

The recipient mice were pre‐treated with gentamicin (1 mg mL^−1^) for two days after being lethally irradiated (9 Gy). Subsequently, they received intravenous injection of a 1:1 mixture of WT (CD45.1) bone marrow cells with bone marrow cells from either *Dap5^flox^* or HO‐*Dap5^ΔFoxp3^* mice (5 × 10^6^ cells total). Chimeras were analyzed 8 weeks after reconstitution.

### Adoptive Transfer of CD4^+^ T Cells

Splenic naïve CD4^+^ T cells were isolated from either *Dap5^flox^* or HO*‐Dap5^ΔFoxp3^* by flow cytometric sorting based on their surface marker (CD4^+^CD25^−^CD44^−^CD62L^+^),^[^ ^68^ ^]^ and then were intravenously injected into *Rag1^−/−^* recipient mice (4 × 10^5^ cells per mouse). In co‐transfer assay, naïve CD4^+^ T cells (4 × 10^5^) isolated from the spleens of WT C57BL/6J mice were mixed with Tregs (CD4^+^CD25^hi^) (2 × 10^5^) isolated from either *Dap5^flox^* or HO*‐Dap5^ΔFoxp3^* mice, and this mixture was then injected into *Rag1^−/−^* recipient mice.

### Subcutaneous Tumor Growth

To generate mouse subcutaneous tumor growth model, Panc02 cells (2 × 10^6^) or MC38(2 × 10^6^) were subcutaneously inoculated into the flank of 6–8‐week‐old *Dap5^flox^* and HE*‐Dap5^ΔFoxp3^* mice. Mice were monitored daily, and tumors measured two or three times weekly by caliper. The tumor volumes were calculated by the formula: tumor volume (mm^3^)  =  0.5 × length × width × width. Mice were euthanized when tumor volumes reached ≈ 2000 mm^3^, or when animals displayed limited locomotion and were moribund.

### In Vivo Puromycin Labeling of Protein Synthesis

When subcutaneous Panc02 tumors reached a volume of ≈500 mm^3^, mice were injected with puromycin (50 mg kg^−1^, Aladdin, #P113126) through the tail vein. After 1 h, mice were euthanized, and tumors were immediately collected and dissociated into single‐cell suspensions. The incorporation of puromycin into newly synthesized proteins within tumor‐infiltrating T cells was quantified by flow cytometry to assess their translational activity.

### Flow Cytometry

Cells (1 × 10^6^) were harvested, washed twice with PBS, and resuspended in staining buffer (PBS containing 3% FBS). Cells were routinely stained with Zombie Aqua to filter dead cells. For surface staining, cells were incubated with antibodies for 30 min at 4 °C in the dark. After staining, cells were washed twice with staining buffer and fixed with fixation buffer (Biolegend, Cat#421401) for 15 min at RT. For intracellular staining, fixed cells were permeabilized using Perm buffer (BD Biosciences, Cat#73162) for 15 min at RT. Intracellular antibodies were then stained in the permeabilization buffer overnight at 4 °C. After staining, cells were washed twice and resuspended in staining buffer for analysis. Data acquisition was performed on a BD LSRFortessa flow cytometer.

### Construction of DAP5 Phylogenetic Tree

The amino acid sequence of human DAP5 was used as the bait to retrieve the DAP5 homologs present in all the possible creatures by querying Non‐Redundant Protein Sequence Database with *blastp* (v. 2.5.0, https://www.ncbi.nlm.nih.gov/books/NBK52640/) as the setting of “*blastp ‐db nr ‐query human_dap5.fa ‐out dap5.blast ‐num_threads 4 ‐outfmt 6 ‐max_target_seqs 100000*”. The obtained 45120 sequences underwent two rounds of filtration: only the sequences with similarity to human DAP5 over 30% and *e*‐value under 0.05 were kept and for multiple sequences belonging to the same species, the one with the highest similarity was preserved. After filtration, DAP5 homologs from 3292 species were aligned with *muscle* (v. 3.8.1551) followed by construction of maximum likelihood‐based phylogenetic tree with *fasttree* (v. 2.1.10).^[^ ^69^, ^70^ ^]^ The tree was visualized with *ggtree* (v. 3.6.2).^[^ ^71^ ^]^

### RNA Immunoprecipitation Sequencing (RIP‐seq) and Data Analysis

Cells were crosslinked in 0.3% formaldehyde solution for 10 min followed by reaction termination in 2 M glycine solution. The cell pellet was washed twice with 1×PBS and then lysed in RIP buffer (25 mM Tris pH 7.4, 150 mM KCl, 5 mM EDTA, 0.2% CA‐630, 0.05% SDS) at 4 °C for 1 h. The cell lysate containing nucleic acid‐protein complexes was sheared by ultrasonication and then centrifuged to obtain the supernatant, one‐tenth of which was aliquoted as input. The remaining supernatant was subjected to immunoprecipitation by adding 5 µg anti‐DAP5 antibody (Santa Cruz, Cat#sc‐374236) conjugated with Dynabeads Protein A (Thermo Fisher, Cat#10001D) and incubation at 4 °C for 16 h. The antibody‐DAP5‐nucleic acid complexes were collected by magnetic stand. Both the immunoprecipitated and input samples were than digested with DNase I (NEB, Cat#M0303S) at 37 °C for 15 min to remove DNA contamination, followed by protein removal by digestion with Proteinase K (NEB, Cat#P8107S) at 55 °C for 30 min. The resulting RNA fragments were purified with phenol: chloroform: isoamyl alcohol (125:24:1) (Sigma‐Aldrich, Cat#P2069) and subjected to library construction with the Next Ultra RNA Library Prep Kit for Illumina (NEB, Cat#E7770S). The libraries were purified by the VAHTS DNA Clean Beads (Vazyme, Cat#N411) and analyzed on Agilent 2100 Bioanalyzer for measuring fragment size distribution (≈ 150 bp). The qualified libraries were sequenced on Illumina Nova Seq 6000.

The raw *fastq* files were checked with *Fastqc* (v. 0.11.9) for quality control. The adapters were removed from the raw reads with *Cutadapt* (v. 1.18) and mapped onto the human genome (hg38) with *STAR* (v. 2.7.1a). The SAM files were sorted with *Samtools* (v. 1.3.1) and the possible PCR duplications were removed with *Picard* (v. 2.23.3). Consequently, *MACS2* (v. 2.1.1.20160309) was applied for peaks‐calling, during which the input samples were used to deduct background noise. The peak annotation and motif analysis were realized with *Homer2* (v. 4.1.5). Comparison between iTreg and naïve CD4^+^ T cells was made by DEseq2 (v. 1.32.0). Peaks with absolute log2Foldchange no less than 1 and adjusted *p*‐value less than 0.05 were designated as significantly different.

### m^6^A‐RNA Immunoprecipitation Sequencing and Data Analysis

Total RNAs were extracted from the cells with Trizol and one‐tenth of the purified RNAs were preserved as input. The remaining RNAs were fragmented into ≈200 bp pieces with the NEBNext Magnesium RNA Fragmentation Module (NEB, Cat#E6150S) and purified with the Monarch RNA Cleanup Kit (NEB, #T2040L). The purified RNA fragments were dissolved in 100 µL IP buffer (RNase free H_2_O containing 1 M Tris‐HCl, pH = 7.4, 10% NP‐40, 5 M NaCl, and 40U RNase inhibitor) and incubated with 5 µg anti‐m^6^A antibody (D9D9W) (CST, Cat#56593) at 4 °C for 2 h. The antibody‐m^6^A‐RNA complexes were collected with Dynabeads Protein A (Thermo Fisher, Cat#10001D) and washed three times with 1×PBS. The clean Protein‐A‐antibody‐m^6^A‐RNA complexes were treated with proteinase K at 55 °C for 30 min to remove potential protein contamination. The m^6^A‐RNAs were then precipitated with phenol: chloroform: isoamyl alcohol (125:24:1) (Sigma‐Aldrich, Cat#P2069) followed by rRNA removal with Ribo‐off rRNA Depletion Kit (Vazyme, Cat#N406‐01). The resultant m^6^A‐RNAs and the aforementioned input samples were purified using RNA Clean beads (Vazyme, Cat#N412‐01) and reverse‐transcribed for library construction with NEBNext Ultra TM RNA Library Prep Kit for Illumina (NEB, Cat#E7770S). The libraries were purified with VAHTS DNA Clean Beads (Vazyme, Cat#N411) and analyzed on Agilent 2100 Bioanalyzer for measuring size distribution. The qualified libraries were then sequenced on Illumina NovaSeq 6000.

The *fastq* files were evaluated with *Fastqc* (v. 0.11.9) for quality control before being mapped onto the human genome (hg38) with *STAR* (v. 2.7.1a). The alignment SAM files were sorted with *Samtools* (v. 1.3.1). The m^6^A signal distribution was identified by *MACS2* (v. 2.1.1.20160309), during which the input samples were used to deduct background noise. The peak annotation and motif analysis were then realized with *Deeptools* (v. 3.3.1). Comparison between iTreg and naïve CD4^+^ T cells was made by DEseq2 (v. 1.32.0). Peaks with absolute log2Foldchange no less than 1 and adjusted p‐value less than 0.05 were designated as significantly different.

### Ribosome Sequencing and RNA Sequencing and Data Analysis

Live cell pellet was gently resuspended with culture medium containing 0.1 mg mL^−1^ CHX (Beyotime, Cat#SC0353) and washed once with 1×PBS containing 0.1 mg mL^−1^ CHX. The clean cell pellet was lysed in 1 mL lysis buffer at 4 °C for 10 min followed by incubation of 10 µL RNase I (BBI, Cat#B600476) at RT for 30 min. The RNAs were extracted by Trizol and rRNA was removed with SP‐ribo‐Pools (Forever Star, Cat#FS‐R1056‐04) followed by clean‐up with VAHTS RNA Clean Beads (Vazyme, Cat#N412). The clean RNA samples were then treated with T4 polynucleotide Kinase (PNK, Vazyme, Cat#N102) to create RNA fragments with 3′‐hydroxyl and a 5′‐phosphate that are compatible with the following deep sequencing library construction. Following PNK treatment, the ribosome‐protected mRNA fragments (RPFs) were purified by VAHTS RNA Clean Beads followed by library construction with VAHTS Small RNA Library Prep Kit for Illumina (Vazyme, Cat#NR801). The libraries were fractioned on 6% PAGE minigel and the bands ranging ≈ 150 bp were recovered and purified for quality control and deep sequencing on Illumina NovaSeq 6000.

The raw *fastq* files were checked with *Fastqc* (v. 0.11.9) for quality control. *Trim‐galore* (v. 0.6.6) is then used to remove sequencing adaptors and low‐quality reads. QC‐passed reads were then subjected to *bowtie* (v. 1.3.0) to remove mycoplasma contamination and mapped to human ribosome sequences in order to remove rRNA reads. Reads unmapped in this step are collected, subjected to *STAR* (v. 2.7.8a) and mapped to human reference genome (hg38). Reads mapped to the transcriptome are collected for subsequent analysis. Transcriptome‐aligned reads were processed with *Ribowaltz* (v. 1.2.0), and differences in ribosome binding were analyzed using *DESeq2* (v. 1.3.0). Function enrichment was performed using *Cluster Profiler* (v. 3.18.1). Translational efficiency (TE) for a certain transcript was determined as below:

(1) $$\mathrm{TE}=\mathrm{FPKM}\left(\mathrm{Ribosome}-\mathrm{profiling}\right)/\mathrm{FPKM}\left(\mathrm{RNA}-\mathrm{seq}\right)$$

### Single Cell RNA Sequencing (scRNA‐seq) and Analysis

Tumor tissues were minced into small pieces and digested in 1 mg mL^−1^ collagenase I (Sigma‐Aldrich) at 37 °C for 40 min with gentle agitation. The resulting cell suspension was filtered through a 70 µm cell strainer to remove debris. Lymphocytes were enriched using a Percoll gradient centrifugation, and CD3^+^ T cells were further isolated using the Stemcell CD3^+^ T Cell Isolation Kit according to the manufacturer's instructions. Thymus tissues were mechanically dissociated and filtered through a 70 µm strainer to obtain single‐cell suspensions. PBMCs were isolated from whole blood by density gradient centrifugation using a Percoll gradient centrifugation. Single cell suspensions with high cell viability were subjected to library construction with the Chromium Next GEM Single Cell 5′ Kit v2 (10x Genomics, Cat#2000149) and Chromium Single Cell V(D)J Reagent Kit (10x Genomics, Cat#100016) according to the manufacture's instructions. Briefly, individual cells were captured and barcoded in Gel Beads in Emulsion (GEMs). The RNA molecules with cell‐specific barcodes were reverse‐transcribed into cDNA within the GEMs. Then cDNA samples were amplified for sequencing library construction by adding sequencing adapters and indexes. TCR‐ and BCR‐ enriched libraries were generated with aliquots from each of the aforementioned cDNAs using the Chromium Single Cell V(D)J Enrichment kit. The quality and quantity of the constructed libraries were evaluated with Agilent 2100 Bioanalyzer. The qualified libraries were sequenced on the Sequencer (Illumina, Novaseq6000).

The off‐machine raw data were pre‐processed using the *Cell Ranger* toolkit (v. 6.2.1). Briefly, the *STAR* aligner integrated in the *cellranger count* command was used to align the reads to the reference transcriptome (mm10). Cell barcodes and unique molecular identifiers (UMIs) were used to quantify gene expression at single‐cell resolution, generating the gene expression matrix for downstream analysis with the R toolkit *Seurat* (v4.0.1).^[^ ^72^, ^73^ ^]^ After further quality control and gene expression normalization, highly variable genes that drove cell‐to‐cell differences were identified. Principle component analysis (PCA) was performed to identify the most significant sources of variation. Uniform Manifold Approximation and Projection (UMAP) was utilized to perform non‐linear dimensionality reduction and visualization. Cells were then clustered based on their gene expression profiles using the algorithm of graph‐based clustering. Cell clusters were annotated based on the expressions of canonical markers. The differentially expressed genes between cell clusters or experimental groups were performed with the function of *FindMarkers(object = xx, ident.1 = ″″, ident.2 = ″″, verbose = FALSE)*.

### Single Cell TCR and BCR Analysis

The single cell V(D)J sequencing data were analyzed with the *cellranger vdj* pipeline to obtain the productive nucleotide sequences and translated amino acid sequences for TCRs (α and β chains) or BCR (H and L chains). Identities of cells with TCR or BCR information were recalled via the unique cellular barcode. Cells with identical TCR_αβ_ pair or BCR_HL_ pair were designated as clonal expanded T/B cells. Indexes of clonal expansion were calculated as previously described.^[^ ^74^ ^]^

### RNA Velocity Analysis

RNA velocity analysis was performed using scvelo (v0.3.3).^[^ ^75^ ^]^ Briefly, spliced and unspliced read counts were quantified with the *velocyto* command line tool (v0.17.17) to generate the loom files from BAM files obtained via Cell Ranger analysis, using the GRCm39 reference genome annotation. The resulting loom files were integrated into the preprocessed Seurat object containing CD8⁺ T cell data. Velocity estimation was then carried out using scvelo functions in the following steps: scvelo.pp.moments, scvelo.tl.velocity, and scvelo.tl.velocity_graph, with the stochastic mode enabled. Finally, trajectory inference was performed using RNA velocity‐based Partition‐based Graph Abstraction (PAGA), a method that estimates connectivity between partitions of the data manifold.^[^ ^76^ ^]^

### Statistical Analysis

Statistical analysis was performed using GraphPad Prism (v. 8.0), R (v. 4.3.2), Microsoft Excel and IBM SPSS Statistics (v. 27). The normality of data distribution was assessed using the Shapiro‐Wilk test. For comparisons between two groups, an unpaired two‐tailed Student's t‐test was employed if the data followed a normal distribution. In cases of non‐normal distribution, the Mann‐Whitney U test was used. Correlations between variables were evaluated using Pearson's correlation coefficient for normally distributed data. Statistical significance was defined as a *p*‐value less than 0.05. All the statistical tests were two‐sided unless otherwise specified.

## Conflict of Interest

The authors declare no conflict of interest.

## Author Contributions

J.W. conceived and supervised this study. C.H. and C.X. co‐supervised this study. X.L. performed most of the experiments, analyzed the data with the help from C.X., X.F., J.Z., Y.L., Z.L., and S.Z. J.W. and G.P. performed the bioinformatic analysis. C.H. arranged the collection and processing of the clinical samples with the help from X.L., J.Z., and H.Y. Y.F. and L.B. provided necessary interpretations for the clinical data. J.W. wrote the manuscript with inputs from rest of the authors.

## Supporting information



Supporting Information

## Data Availability

The study is compliant with the “Guidance of the Ministry of Science and Technology (MOST) for the Review and Approval of Human Genetic Resources”. All relevant approvals related to the export of genetic information and any sequencing data relevant to this work were obtained from Ministry of Science and Technology of the People's Republic of China (Approval No.: 2024KS269). The raw sequence data reported in this paper have been deposited in the Genome Sequence Archive (Genomics, Proteomics & Bioinformatics 2021) in National Genomics Data Center, China National Center for Bioinformation/Beijing Institute of Genomics, Chinese Academy of Sciences (GSA: CRA029673) that are accessible at https://ngdc.cncb.ac.cn/gsa.
