# Supplementary material for: Targeting DAP5 Disrupts Alternate Mode of Translational Initiation in Tregs and Potentiates Antitumor Immunity
Source: Adv Sci (Weinh). 2025 Dec 28;13(10):e20625. doi: 10.1002/advs.202520625 (PMC12915102; doi:10.1002/advs.202520625)
Supplement: Supplementary file 1 — Supporting Information [file ADVS-13-e20625-s001.pdf]

Supplementary Materials for

Targeting DAP5 disrupts alternate mode of translational initiation in Tregs and potentiates antitumor immunity

Lai *et al*.

Correspondence to:

xiechengme.i@163.com (C.X.), richard-hc@hotmail.com (C.H.), junwang2023@sjtu.edu.cn (J.W.)

This PDF file includes:

Supplementary Text

Figure. S1 to S7

Table S1 to S6

Movie S1

Supplemental figures and figure legends

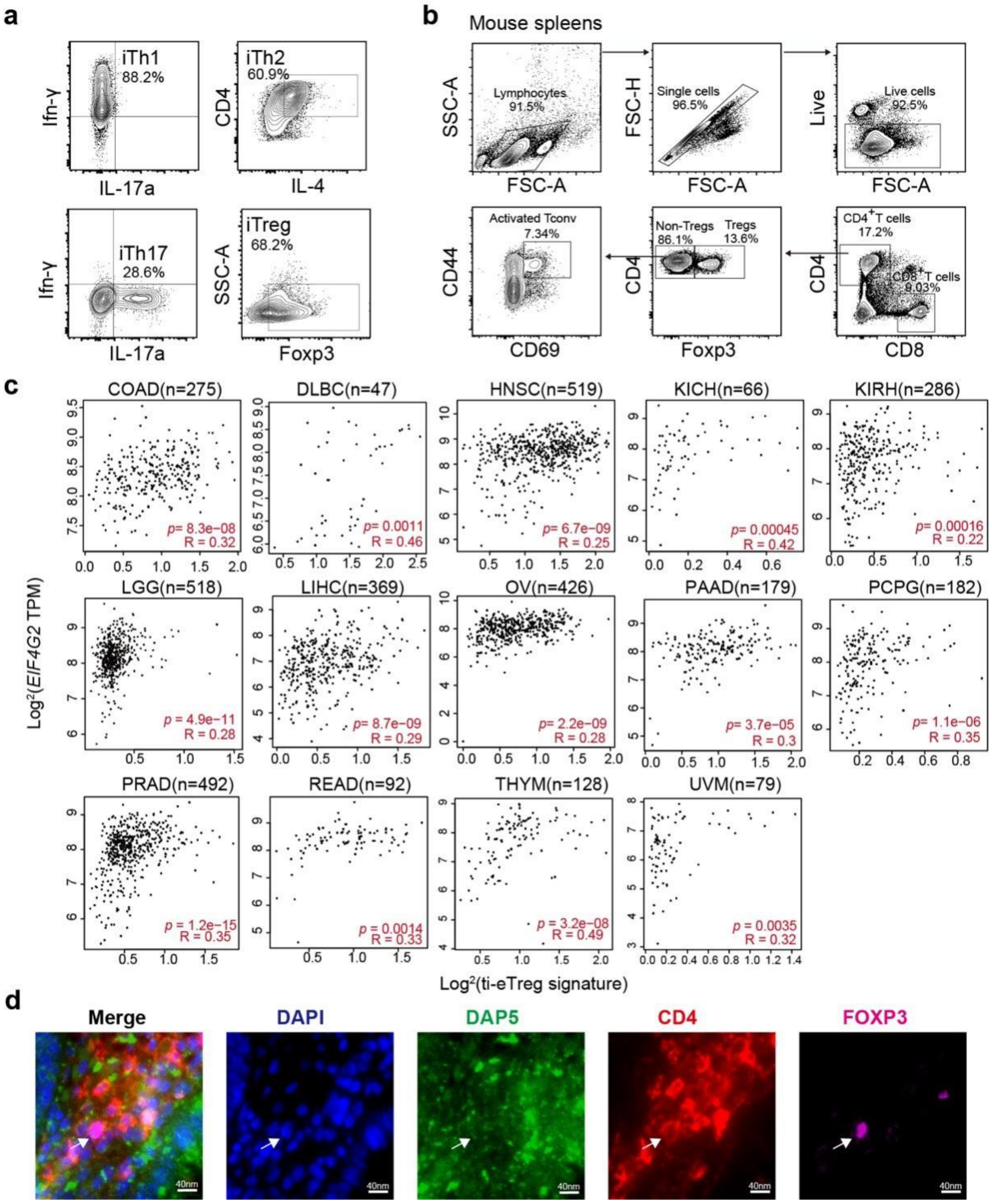

Figure S1. CDT is suppressed in Tregs. a) Representative flow cytometric plots for iTh1, iTh2, iTh17 and iTreg. **b)** Gating strategy for activated Tconv cells ($CD4^+Foxp3^-CD44^+CD69^+$) from mouse spleens. **c)** Scatter plots showing positive correlations between *EIF4G2/DAP5* expression and effector Treg signature across 14 cancer categories. Each dot represents an individual tumor specimen. We used the non-log scale for calculation and used the log-scale axis for visualization. Note: colorectal adenocarcinoma (COAD), diffuse large B-cell lymphoma (DLBC), head and neck squamous cell carcinoma (HNSC), kidney chromophobe (KICH), kidney renal clear cell carcinoma (KIRH), lower grade glioma (LGG), liver hepatocellular carcinoma (LICH), ovarian serous cystadenocarcinoma (OV), pancreatic adenocarcinoma

(PAAD), pheochromocytoma and paraganglioma (PCPG), prostate adenocarcinoma (PRAD), rectum adenocarcinoma (READ), thymoma (THYM), uveal melanoma (UVM). **d)** Representative mIF images of $CD4^{+}Foxp3^{+}DAP5^{+}$cells in human CRC tissues.

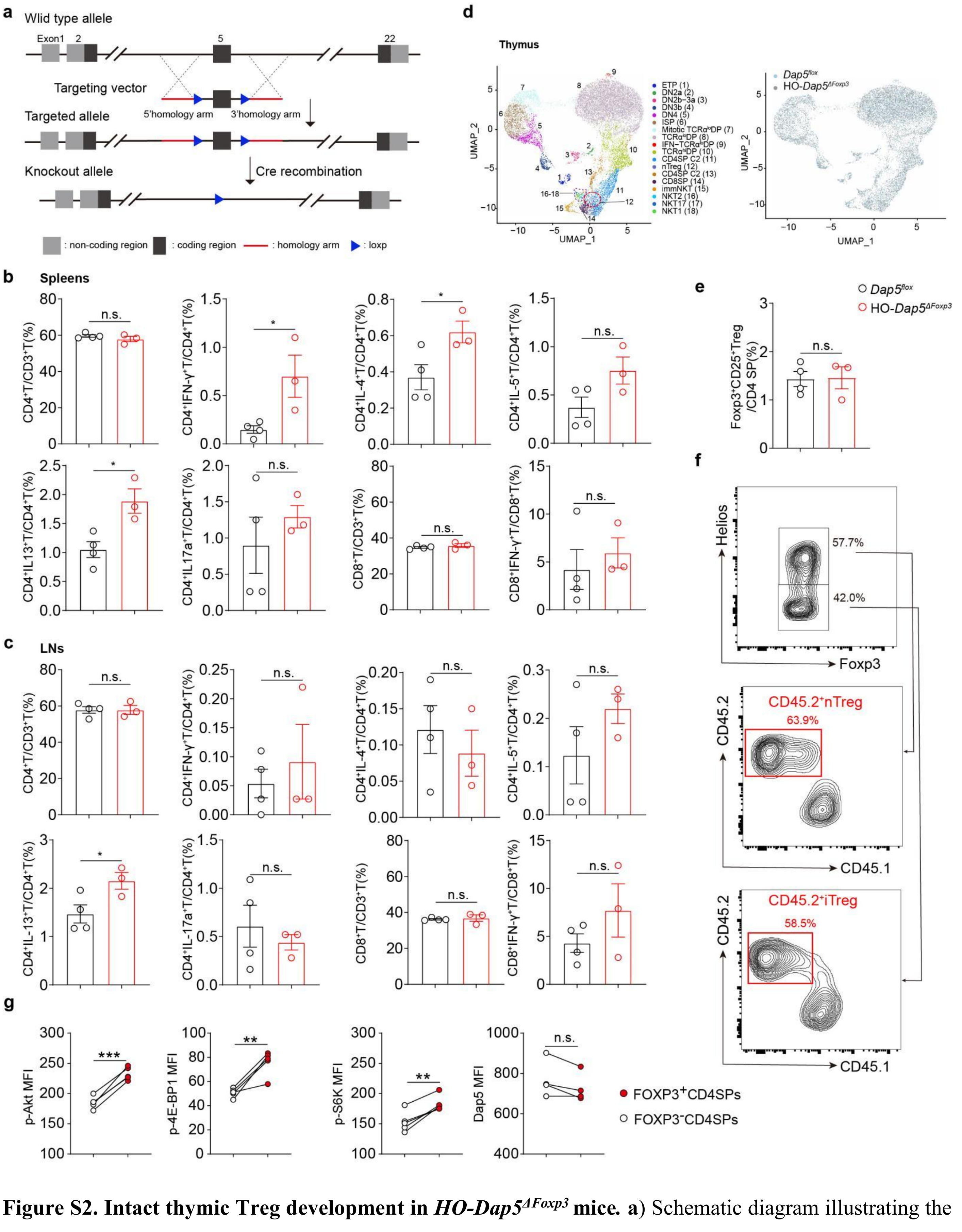

Figure S2. Intact thymic Treg development in *HO-Dap5$^{\Delta Foxp3}$* mice. a) Schematic diagram illustrating the construction of the *Dap5$^{\Delta Foxp3}$* mouse strain. **b and c)** Bar plots showing the proportions of indicated T cell subpopulations in the spleens (**b**) and LNs (**c**) of 4-weeks-old *Dap5^{flox}* and age-matched *HO-Dap5$^{\Delta Foxp3}$* mice.

d) 2D-UMAP map displaying the distribution of thymocyte subpopulations determined by scRNA-seq. **e**) The bar plots showing that *Dap5*^{flox} and *HO-Dap5*$^{\Delta Foxp3}$ mice had similar proportions of tTregs in thymus. **f**) Gating strategy of CD45.2^{+} Treg deriving from *Dap5*^{flox} or *HO-Dap5*$^{\Delta Foxp3}$ donor mice in CD45.1^{+} recipient in bone marrow chimerism experiment. **g)** Flow cytometric results showing increased phosphorylation levels of Akt, S6K and 4E-BP1 but unchanged Dap5 expression in Foxp3^{+}CD4SPs than in Foxp3^{-}CD4SPs from mouse thymus. *P*-values were determined by two-tailed student's *T*-test (**b**, **c**, **e, g**), *p<0.05. ETP: Early T cell progenitors; DN: Double negative thymocytes; ISP: Immature single (CD8^{+}) positive thymocytes; SP: Single positive thymocytes; immNKT: immature natural killer T cells.

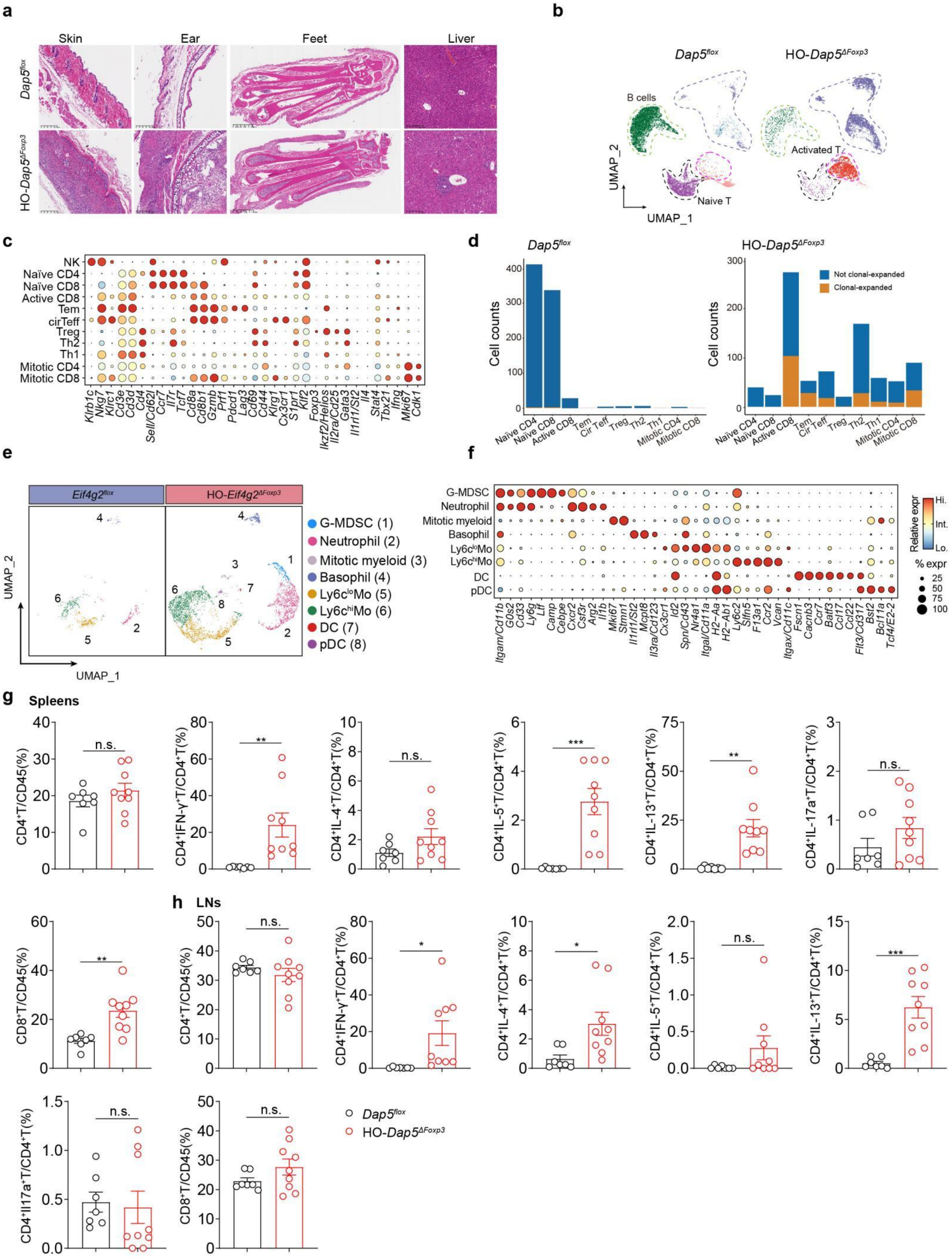

Figure S3. Lethal autoinflammatory responses in *HO-Dap5$^{\Delta Foxp3}$* mice. a) H&E staining showing elevated inflammations in the tissues of skin, ear, feet, and liver from the sick *HO-Dap5$^{\Delta Foxp3}$* mice at the age

of 8-weeks. **b**) 2D-UMAP map displaying the distribution of T, B and myeloid cells in PBMCs from sick *HO-Dap5*$^{\Delta Foxp3}$ mice and age-matched *Dap5*^{flox} mice. **c**) Dot plots showing marker gene expressions across T cell subpopulations and NK cells. **d**) Stacked bar plots depicting the compositions of clonal-expanded and non-expanded T cells across subpopulations between *Dap5*^{flox} and *HO-Dap5*$^{\Delta Foxp3}$ mice. **e**) Distribution of subpopulations of myeloid cells on the 2D-UMAP map at single cell resolution. **f**) Dot plots showing marker gene expressions across myeloid cell subpopulations. **g** and **h**) Dar plots comparing proportions of indicated inflammatory T cell subpopulations in the spleens (**g**) and LNs (**h**) between 6-weeks-old HO-*Dap5*$^{\Delta Foxp3}$ mice and age-matched *Dap5*^{flox} mice. *P*-values were determined by two-tailed student's *T*-test (**g** and **h**), *$p<0.05$, **$p<0.01$, ***$p<0.001$.

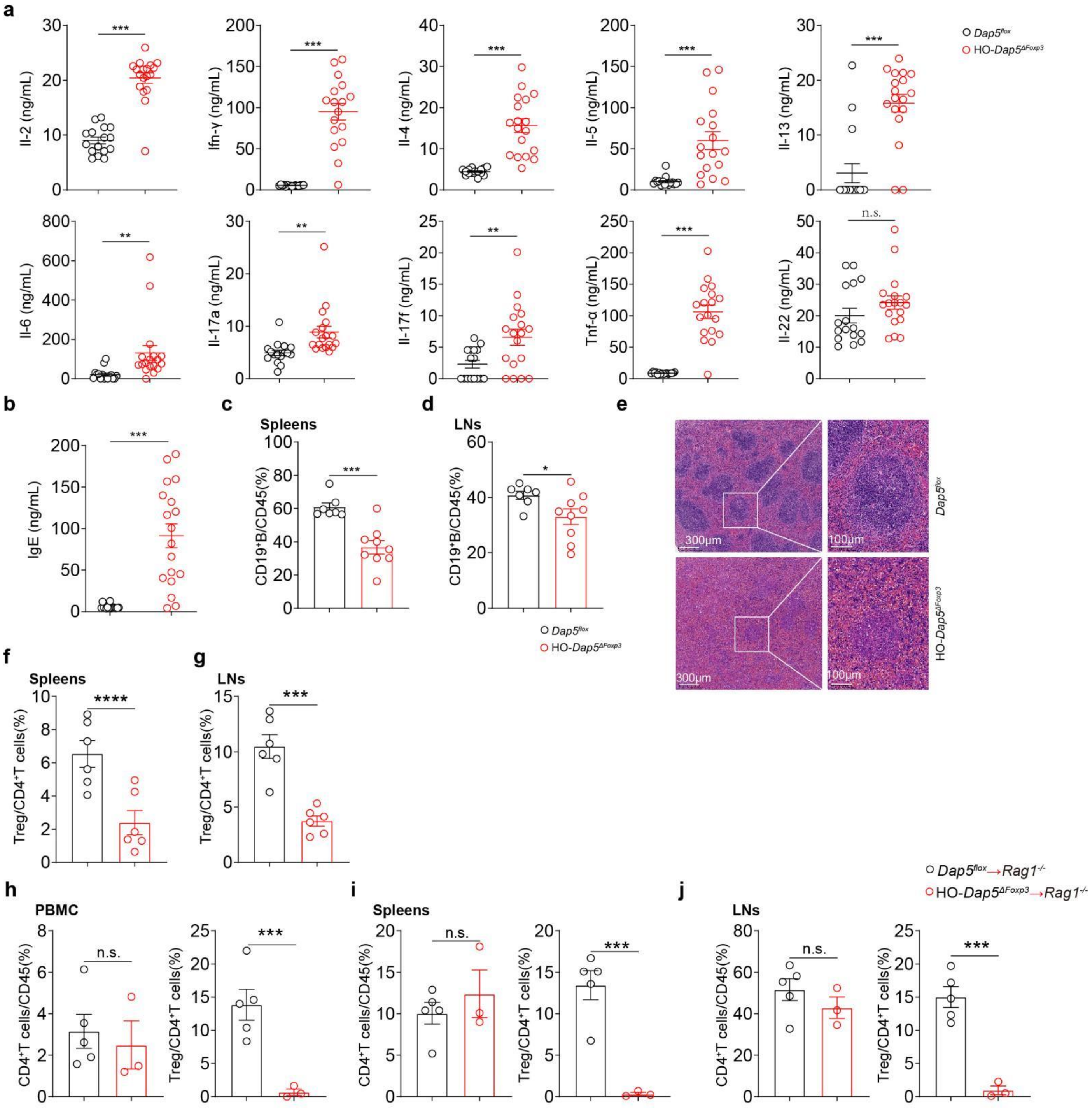

Figure S4. Tregs with homozygous deletion of *Dap5* displayed survival defects in the periphery. a) Significantly elevated concentrations of inflammatory cytokines in the peripheral blood of HO-*Dap5*$^{\Delta Foxp3}$ mice. The cytokine concentrations were determined by the Inflammatory Cytokine Cytometric Bead Array (CBA) kit with plasma samples. **b**) Significant elevation of IgE levels in the plasma of *HO-Dap5*$^{\Delta Foxp3}$ mice. IgE concentrations were determined by Elisa assay. c and d) The bar graph shows a significant reduction of B cells in the spleens and lymph nodes of *HO-Dap5*$^{\Delta Foxp3}$ mice. **e**) H&E staining showing destroyed GC structures in the spleens of sick HO-*Dap5*$^{\Delta Foxp3}$ mice. f and g) Bar plot indicating decreased frequencies of

peripheral Treg cells in *HO-Dap5*$^{\Delta Foxp3}$ mice compared to *Dap5*^{flox} mice. h, i and j) The bar plots showing the percentage of CD4^{+} T and Treg cells derived from naïve CD4^{+} T cells of HO-*Dap5*$^{\Delta Foxp3}$ or *Dap5*^{flox} mice in PBMC, spleens and LNs of the *Rag1*$^{-/-}$ recipient mice. *P*-values were determined by two-tailed student's *T*-test (**a, b, c, d, f, g, h, i** and **j**), *$p<0.05$, **$p<0.01$, ***$p<0.001$, ****$p<0.0001$.

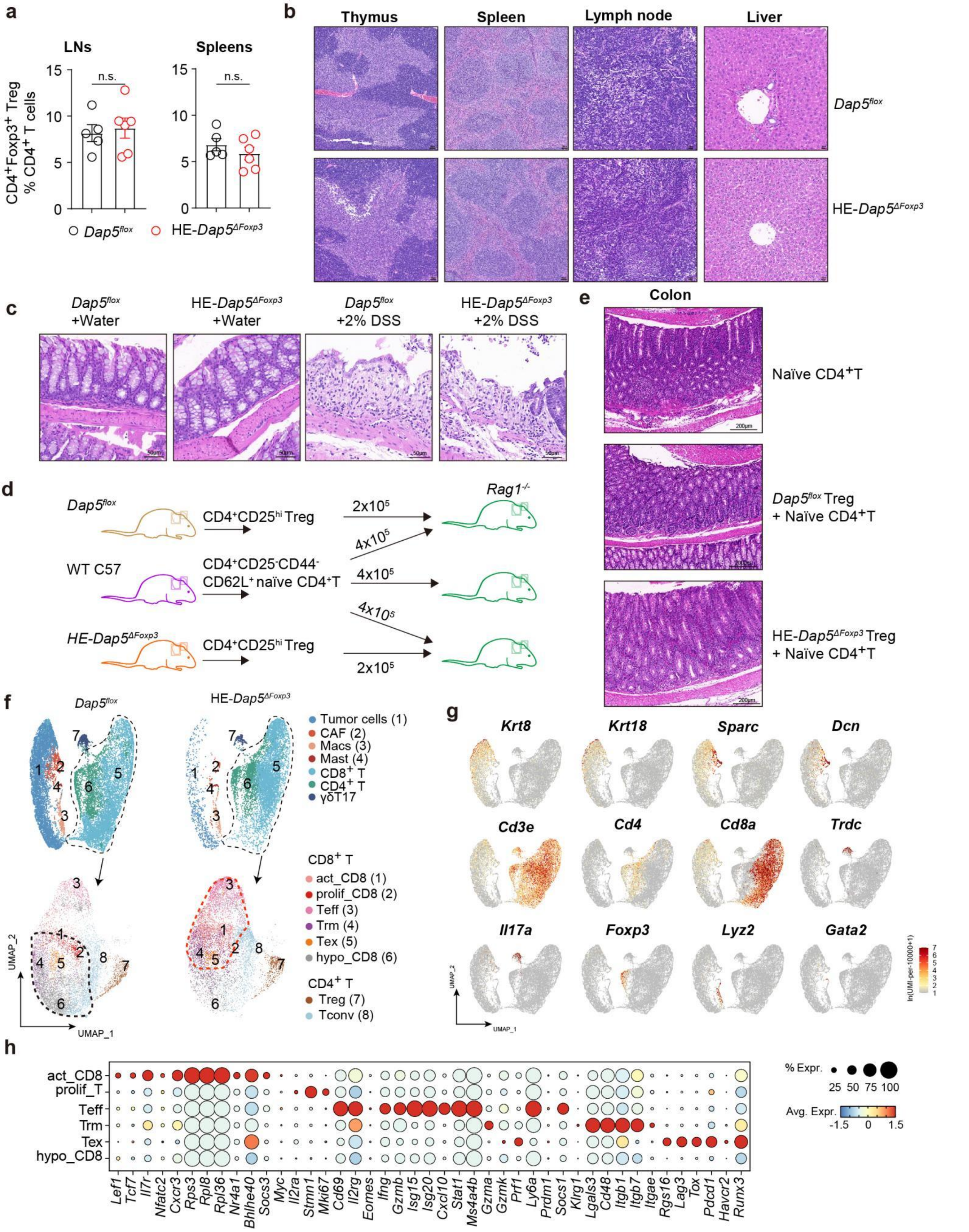

Figure S5. Mice with haploinsufficiency of Dap5 in Tregs are defective to maintain immunosuppressive TME. a) The bar plots showing that *Dap5*^{flox} and HE-*Dap5*$^{\Delta Foxp3}$ mice had similar proportions of Tregs in LNs and spleens. **b**) H&E staining showed normal structure of thymus, spleen,

lymph node and liver from *Dap5*^{flox} and HE-*Dap5*$^{\Delta Foxp3}$. **c)** Representative histopathological images of colon tissue sections stained with H&E in DSS-induced colitis model. **d)** Schematic diagram depicting the workflow of adoptive T cell transfer induced colitis model. **e)** Representative histopathological images of colon tissue sections stained with H&E in adoptive T cell transfer induced colitis model. **f**) UMAP visualization showing distinct clusters of T-cell subsets and tumor cells within the tumor microenvironment of *Dap5*^{flox} and HE-*Dap5*$^{\Delta Foxp3}$ mice. **g)** Feature plots showing marker gene expressions in cells from subcutaneous tumors from *Dap5*^{flox} and HE-*Dap5*$^{\Delta Foxp3}$. **h**) Dot plots showing marker gene expressions across $CD8^+$ T subpopulations. *P*-values were determined by two-tailed student's *T*-test (**a**), *$p<0.05$.

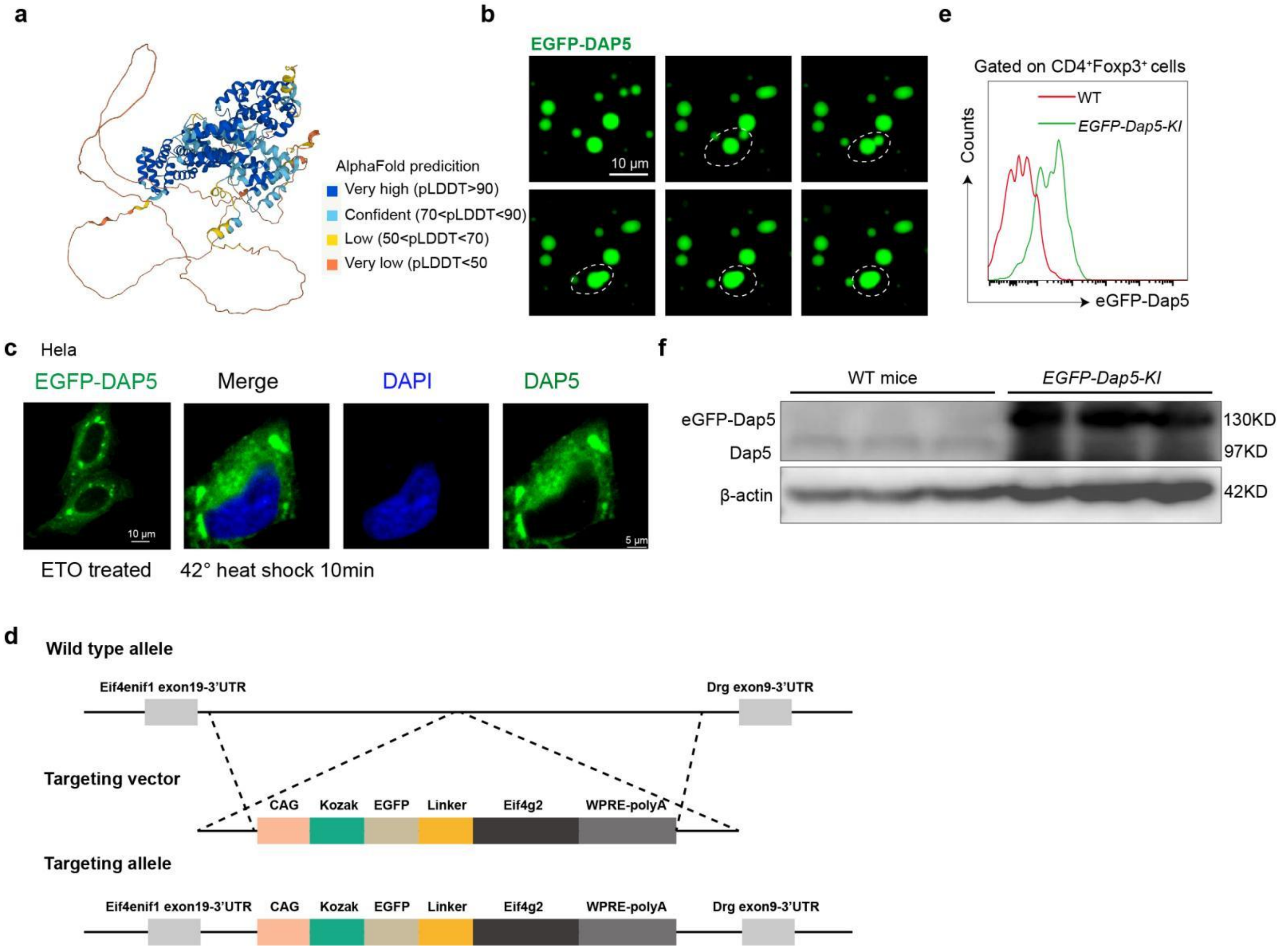

Figure S6. DAP5 undergo LLPS in Tregs. **a**) Prediction of disordered regions in DAP5 using AlphaFold. **b**) The purified recombinant eGFP-DAP5 exhibited dynamic fusion and fission behavior in a solution containing 100 mM NaCl, 20 mM Tris-HCl pH 7.5, and 5% PEG8000. **c**) ETO (50 μM) stimulation or heat shock (42° for 10 minutes) induced the formation of eGFP-DAP5^{+} puncta in HeLa cells expressing eGFP-DAP5. Images were taken under fluorescence microscopy. **d**) Schematic diagram illustrating the construction of the *EGFP-DAP5-KI* mouse strain. **e**) Flow cytometric histogram reconfirming expression of eGFP-DAP5 in the splenic Tregs from the *EGFP-DAP5-KI* mice. **f**) WB demonstrating eGFP-Dap5 expression in the splenic cells from the *EGFP-DAP5-KI* mice.

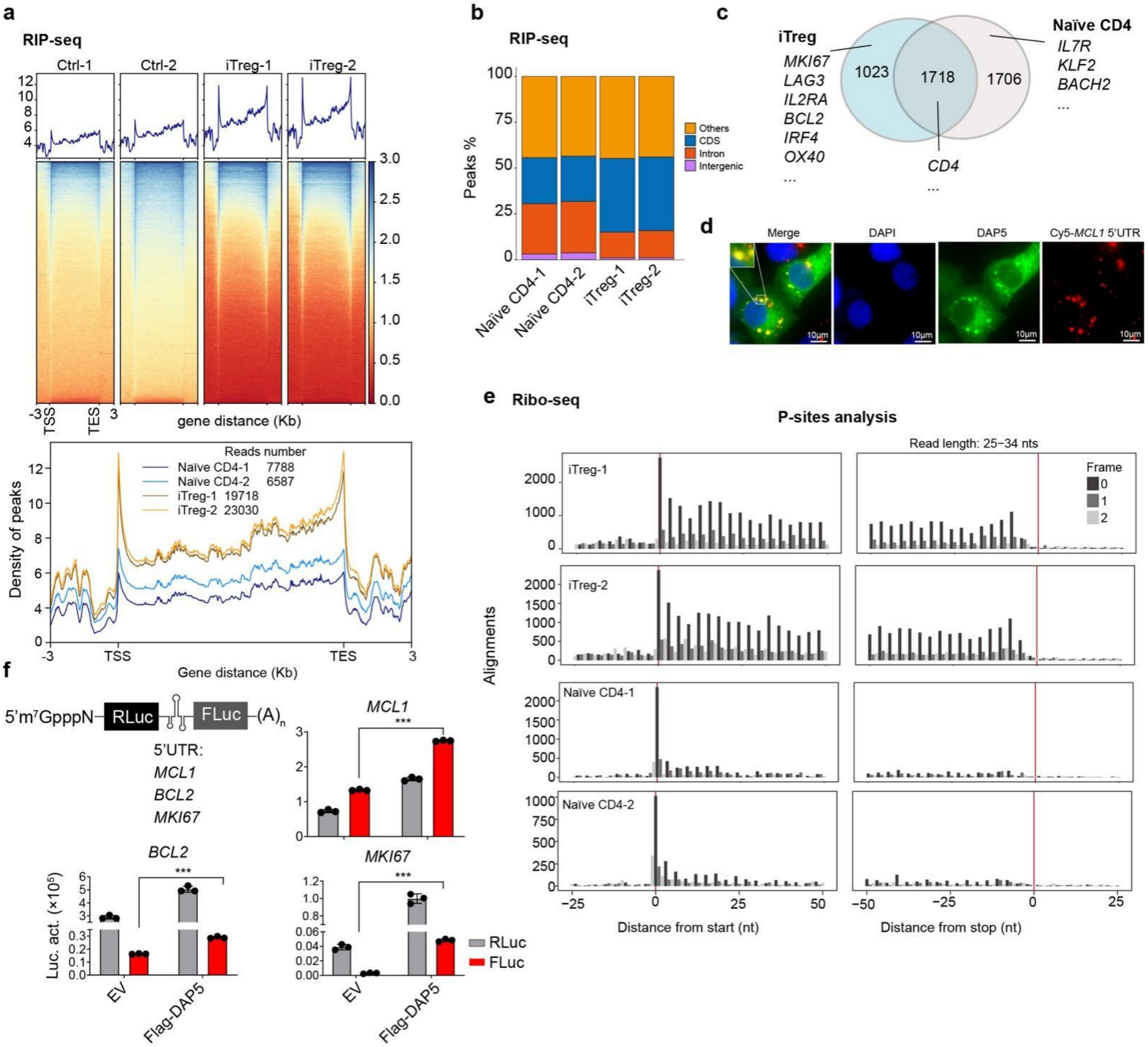

Figure S7. Analysis of RIP-seq and Ribo-seq on human iTregs. a) After annotating DAP5-bound peaks using Homer, profile heatmaps were generated to illustrate the enrichment of the DAP5-bound peaks within the pan-genomic region encompassing 3 kb upstream of the transcription start sites (TSS), gene body and 3 kb downstream of the transcription end sites (TES). **b)** Stacked bar plot showing the composition of DAP5-bound peaks according to their genomic locations in each sample. **c)** Venn diagram summarizing genes whose transcripts had DAP5-binding events unique in either iTregs or naïve $CD4^+$ T cells or in both cell types. **d**) Cy5-labeled 5'UTR of *MCL1* were transfected into HeLa cells expressing eGFP-DAP5. After heat shock at 42° for 10 minutes, distributions of eGFP-DAP5 and Cy5-labeled 5'UTR of *MCL1* were subsequently visualized under fluorescence microscopy. **e**) Bar plots visualizing the distribution of the

ribosome-protected fragments (RPFs) around the P-site in each sample, indicating efficiency of translation initiation and elongation. P-site refers to the peptidyl-tRNA binding site on the ribosome where the growing polypeptide chain is located during translation. The P-site corresponds to the second nucleotide of the codon being decoded by the ribosome. **f**) Construction of a bicistronic expression vector (top left); overexpression of DAP5 in HEK293T cells enhances the expression of FLuc driven by the 5'UTRs of *MCL1, BCL2* and *MKI67*. *P*-values were determined by two-tailed student's *T*-test(F), ***p<0.001.

Supplementary table 1 Clinical characteristics of the cohort of patients with colorectal cancers (CRCs)

CRCs	/	101 (100%)
Paired paracancerous tissues	/	79 (78.22%)
Gender	Male	50 (49.5%)
	Female	51 (50.5%)
Age (mean±SD)	/	67.88±9.83
Survival (mean±SD)	/	59.68±35.51
Average size(cm^3) (mean±SD)	/	33.61±29.06
AJCC7 Stage (%)	Stage I	6 (5.94%)
	Stage II	55 (54.46)%
	Stage III	39 (38.61%)
	Stage IV	1 (0.99%)
Tumor Grade (%)	I	1 (0.99%)
	I-II	22 (21.78%)
	I-III	0 (0%)
	II	62 (61.39%)
	II-III	11 (10.89%)
	III	5 (4.95%)
T Stage (%)	T1	1 (0.99%)
	T2	5 (4.95%)
	T3	75 (74.26%)
	T4	19 (18.81%)
	Unknow	1 (0.99%)
N Stage (%)	N0	61 (60.40%)
	N1	30 (29.70%)
	N2	10 (9.90%)
M Stage (%)	M0	100 (99.01%)
	M1	1 (0.99%)

Supplementary table 2 Marker genes for subpopulations of thymic T cells.

Supplementary table 3 Markers genes for T cell subpopulations in PBMC.

Supplementary table 4 Markers genes for myeloid cell subpopulations in PBMC.

Supplementary table 5 List of antibodies

Antibody	Fluorochrome	Source	Cat #	Dilution
Anti-mouse CD45	AF700	Biolegend	147716	1:200
Anti-mouse CD4	BV421	Biolegend	100543	1:200
Anti-mouse CD8a	BV605	Biolegend	100743	1:200
Anti-mouse IFN-γ	PE	Biolegend	505808	1:200
Anti-mouse FOXP3	AF647	Biolegend	320013	1:200
Anti-mouse IL-4	BV605	Biolegend	504125	1:200
Anti-mouse IL-17A	Percp-cy5.5	Biolegend	506920	1:200
Anti-mouse IL-13	PE	Thermo Fisher	12-7133-81	1:200
Anti-mouse IL-5	APC	Biolegend	504306	1:200
Anti-mouse CD25	FITC	Biolegend	101907	1:200
Anti-mouse CD45.1	PE	Biolegend	110707	1:200
Anti-mouse CD45.2	FITC	Biolegend	109805	1:200
Anti-mouse CD62L	PE/Cy7	Biolegend	104418	1:200
Anti-mouse CD25	PE	Biolegend	102008	1:200
Anti-mouse CD44	APC	Biolegend	103012	1:200
Zombie Aqua fixable viability kit		Biolegend	423101	1:200
PI		Biolegend	421301	1:200
Anti-mouse Helios	PE/Cy7	Biolegend	137236	1:200
7-AAD Viability		Biolegend	420403	1:200
Anti-human CD4	BV421	Biolegend	344632	1:200
Anti-human CD8	PE/Cy7	BD	557746	1:200
Anti-human CD127	PE/Dazzle 594	Biolegend	351336	1:200
Anti-human CD25	PE/Cy7	Biolegend	356108	1:200
Anti-mouse/human Cleaved Caspase-3	PE	CST	12768S	1:200
DAP5 antibody	/	Santa Cruz	sc-374236	/
Puromycin antibody	/	abclonal	A23057	/
MCL-1 antibody	/	Abcam	ab32087	/
CD3 antibody	/	Abcam	ab135372	/
CD19 antiboody	/	CST	90176T	/
β-actin	/	Abcam	ab6276	/
Goat Anti-Rabbit IgG H&L (HRP)	/	Abcam	ab6721	/
Goat Anti-Mouse IgG H&L (HRP)	/	Abcam	ab6789	/
Goat Anti-Mouse IgG H&L (FITC)	/	Abcam	ab150113	/
Goat Anti-Rabbit IgG H&L (AF647)	/	Abcam	ab150079	/

Supplementary table 6 List of primers

Gene name	Forward	Reverse
MKI67	5’-ACGCCTGGTTACTATCAAAAGG-3’	5’-CAGACCCATTTACTTGTGTTGGA-3’
TOP2A	5’-ACCATTGCAGCCTGTAAATGA-3’	5’-GGGCGGAGCAAAATATGTTCC-3’
LAG3	5’-GCGGGGACTTCTCGCTATG-3’	5’-GGCTCTGAGAGATCCTGGGG-3’
OX40	5’-GCAATAGCTCGGACGCAATCT-3’	5’-GAGGGTCCCTGTGAGGTTCT-3’
MCL1	5’-TGCTTCGGAAACTGGACATCA-3’	5’-TAGCCACAAAGGCACCAAAAG-3’
BCL2	5’-GGTGGGGTCATGTGTGTGG-3’	5’-CGGTTCAGGTACTCAGTCATCC-3’
HSPA1A	5’-CCTGGGTGGGGAGGACTTTGAC-3’	5’-CACGGCTCGCTTGTTCTGGCTG-3’
HSPA1B	5’-TTTGAGGGCATCGACTTCTACA-3’	5’-CCAGGACCAGGTCGTGAATC-3’
IL2RA	5’-GTGGGGACTGCTCACGTTC-3’	5’-CCCGCTTTTTATTCTGCGGAA-3’

Supplementary vedio 1 Three-dimensional reconstruction showing the distribution of eGFP-Dap5$^+$ puncta in iTregs derived from the *eGFP-DAP5*-KI mice.
